# Function-specific and Enhanced Brain Structural Connectivity Mapping via Joint Modeling of Diffusion and Functional MRI

**DOI:** 10.1038/s41598-018-23051-9

**Published:** 2018-03-16

**Authors:** Shu-Hsien Chu, Keshab K. Parhi, Christophe Lenglet

**Affiliations:** 1 Electrical and Computer Engineering Department, University of Minnesota, Minneapolis, 55455 USA; 2 Center for Magnetic Resonance Research, University of Minnesota, Minneapolis, 55455 USA

## Abstract

A joint structural-functional brain network model is presented, which enables the discovery of function-specific brain circuits, and recovers structural connections that are under-estimated by diffusion MRI (dMRI). Incorporating information from functional MRI (fMRI) into diffusion MRI to estimate brain circuits is a challenging task. Usually, seed regions for tractography are selected from fMRI activation maps to extract the white matter pathways of interest. The proposed method jointly analyzes whole brain dMRI and fMRI data, allowing the estimation of complete function-specific structural networks instead of interactively investigating the connectivity of individual cortical/sub-cortical areas. Additionally, tractography techniques are prone to limitations, which can result in erroneous pathways. The proposed framework explicitly models the interactions between structural and functional connectivity measures thereby improving anatomical circuit estimation. Results on Human Connectome Project (HCP) data demonstrate the benefits of the approach by successfully identifying function-specific anatomical circuits, such as the language and resting-state networks. In contrast to correlation-based or independent component analysis (ICA) functional connectivity mapping, detailed anatomical connectivity patterns are revealed for each functional module. Results on a phantom (Fibercup) also indicate improvements in structural connectivity mapping by rejecting false-positive connections with insufficient support from fMRI, and enhancing under-estimated connectivity with strong functional correlation.

## Introduction

**Figure 1:**
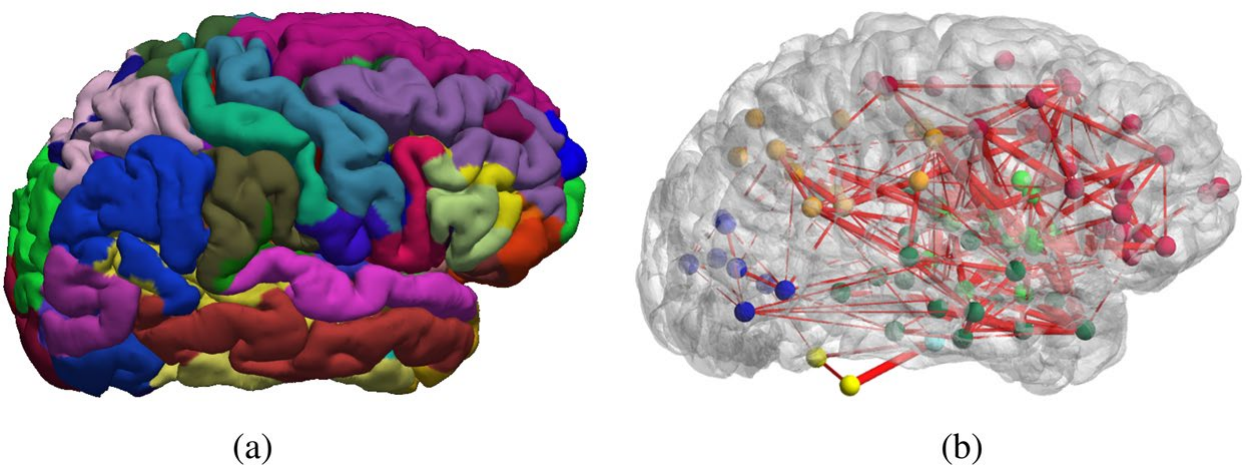
A brain network characterizes the connectivity of regions of interest (Panel (a)) by measuring, for example, the number of tractography streamlines between a pair of regions, or the correlation coefficients between fMRI signals at these locations (Panel (b)). The cortical parcellation (Panel (a)) was generated from a representative HCP dataset using FreeSurfer [http://surfer.nmr.mgh.harvard.edu/]. In this work, all network representations, such as shown in Panel (b), are generated using BrainNet^22^.

The brain can be modeled as a network where nodes represent cortical or sub-cortical gray matter areas, and edges model relationships (connectivity) between nodes^1–3^, as shown in Fig. 1. Gray matter areas are responsible for information (e.g., motor, visual, auditory, language) processing and are physically interconnected via axonal pathways in the white matter, thereby producing functional networks of cortical/sub-cortical areas associated to specific tasks. A network with edges characterized by properties related to white matter pathways is defined as a structural network. On the other hand, if the edges are characterized by statistical relationships from functional signals such as fMRI, electro-encephalogram (EEG) or magneto-encephalogram (MEG), the network is generally referred to as a functional network. By analogy with an electrical circuit that incorporates wiring, components and functionalities, a brain circuit is defined by the anatomical (sub-)network that is associated with a specific function. The mapping of brain functions using either functional or structural networks has been widely investigated^1,2,4–8^. Recently, an increasing number of multimodal explorations, especially combining fMRI and dMRI data, has been conducted to improve our understanding of brain mechanisms, and interactions between functional and structural networks^9–21^.

Significant advances in multimodal imaging techniques, analysis, and modeling have been made to investigate brain changes or group differences^23,24^. In most brain networks studies, the edges’ weights are defined based on either structural or functional connectivity information. However, structural connectivity estimation can be especially challenging in white matter areas with complex fiber orientations (e.g., fiber crossings)^25^. As a result, tractography is prone to false-negative and false-positive results, either under-estimating or generating spurious connections. It is particularly susceptible to missing “weak” long-range connections^26,27^. Conversely, functional connectivity may exist between nodes that are directly or indirectly connected. This can happen when synchronous activity arises between anatomically distinct regions, possibly driven by common sources^28^. Therefore, multimodal approaches may improve the estimation of brain connectivity by combining the strengths of each individual modality (dMRI and fMRI)^10^. We distinguish here three categories of multimodal methods: (i) *Integrative methods*, which use results from one modality to guide connectivity mapping with the other modality, (ii) *Joint analysis methods*, which merge information from independently estimated functional and structural connectivity networks, and (iii) *Joint modeling methods*, which simultaneously model fMRI and dMRI data and estimate either a single combined brain network describing both anatomical and functional connectivity patterns, or separately improved functional and anatomical networks.

In integrative approaches, to assist tractography reconstruction, fMRI is utilized to identify seed regions and refine anatomical parcellation^29–34^. Functional activation maps can be integrated with tractography in order to group and validate estimated white matter pathways^35–38^. Conversely, anatomical connectivity can be employed to predict and penalize the estimation of functional connectivity. Brain activity indeed is intricately linked to its structural connectivity patterns, and functional connectivity is significantly shaped by structural patterns^13–16^. As a result, brain regions with high structural connectivity tend to exhibit high functional connectivity^23^ (but the converse is not necessarily true). Thus, dMRI can be used to support functional connectivity estimation^39,40^. For instance, by considering functional networks as Gaussian graphical models, covariance weighting factors can be defined based on the associated structural connectivity weights^17,18^. Additionally, new metrics such as anatomical-weighted functional connectivity (awFC)^41^ and track-weighted functional connectivity (TW-FC)^42^ were proposed to enhance functional and structural connectivity mapping. Tractography-driven functional connectivity mapping has also been proposed to explore subject-specific interactions between structure and function^43^.

In joint analysis methods, structural and functional results are produced independently and subsequently merged to perform population studies, regression analysis, correlation or multivariate analysis of variance. For example, mean anisotropy (MA) and functional connectivity (from correlation) can be first independently computed from dMRI and fMRI, and then compared^19^. At the network level, joint independent component analysis (ICA) of mixed data from fMRI and dMRI was introduced to explore the connections between white matter microstructure and the default mode network^44^. In addition, theoretical network analysis and dependence studies between functional and structural data were conducted to investigate structural-functional relationships^14,20^. Functional connectivity has also been modeled as a linear by-product of anatomical connectivity^21^.

Unlike joint analysis, a joint modeling approach (as introduced here) simultaneously model fMRI and dMRI data to generate a more complete description of a brain network. We show that this leads to improved estimation of individual networks, and to a novel type of integrated network. In a previous study^45^, structural and functional connectivity parameters were modeled as joint random variables of a Gaussian Mixture Model (GMM). The expectation-maximization (EM) algorithm was employed to recover the underlying probability parameters from the independently generated networks. However, limited spatial continuity (because of independently formulated GMMs across connections), and relatively simple distribution assumptions for anatomical connectivity estimation (e.g., non-Gaussian error caused by fiber crossing) may have restricted the effectiveness of this approach to enhance connectivity mapping results.

**Figure 2:**
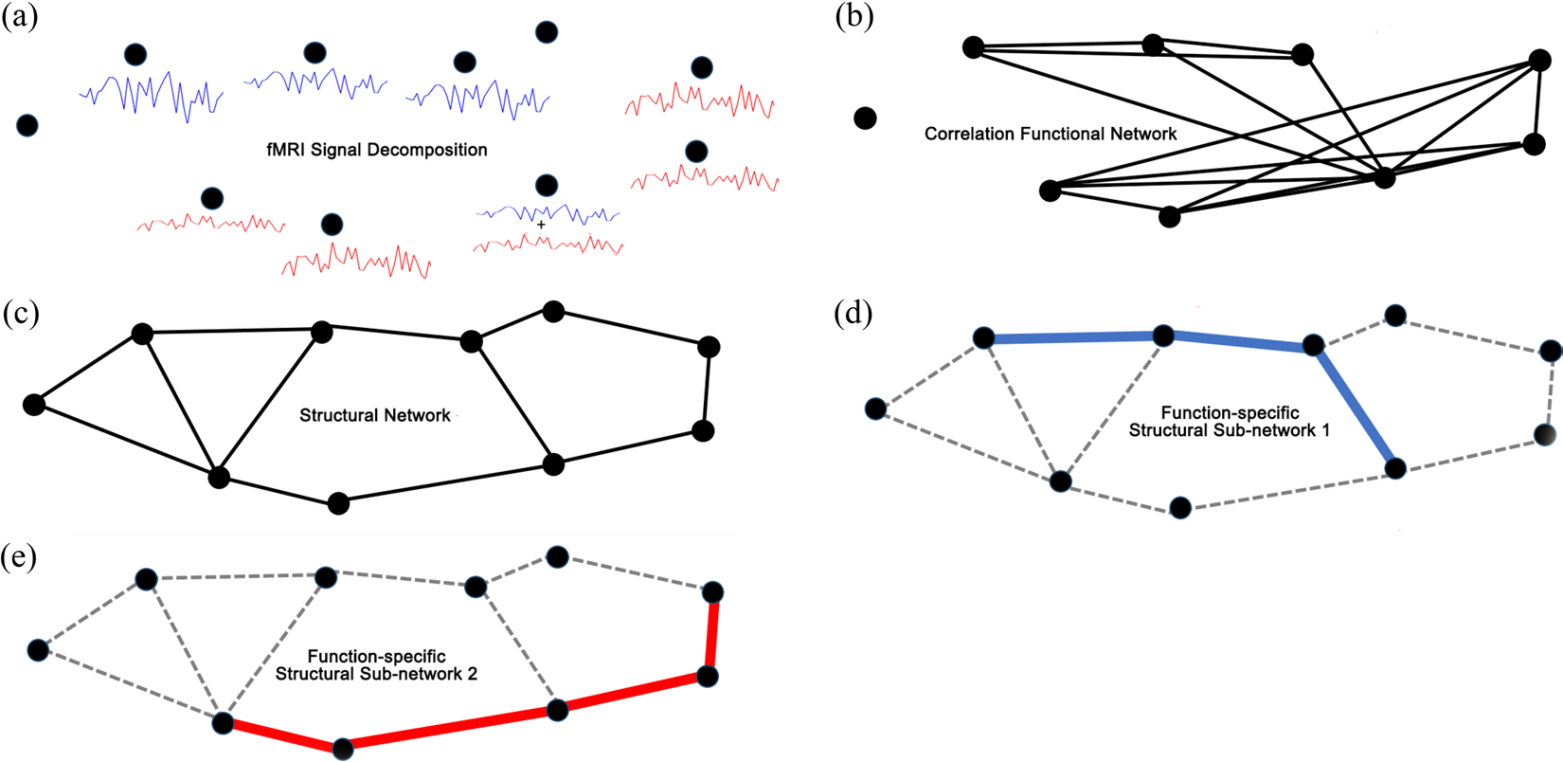
The fMRI signal can be decomposed and represented using basis functions (e.g., ICA components, or “modes”) such as the red and blue signals in panel (a). As shown in panel (b), since regions involved in the same brain function display similar fMRI time courses (blue or red), strong correlations will identify function-specific circuits. Panel (c) shows an example of possible underlying structural network which can support both red and blue “functions”. The proposed joint model combines information from functional and structural connectivity, and can extract the full anatomical sub-networks associated with each function (Panels (d) and (e)).

In this paper, a novel structural-functional brain network model is presented to extract function-specific brain circuits, as illustrated in Fig. 2, and improve anatomical connectivity mapping, by considering system-wide neural communication as a routing problem^46^. The network edges represent white matter pathways carrying electro-chemical signals between nodes (cortical/sub-cortical areas), which we call *information flow*. Edges transmit the information shared among nodes. The nodal activities, corresponding to the received/sent information, are modeled using fMRI activation maps^47^. Therefore, the causal relationship between structural and functional connectivity patterns can be characterized: The absence of anatomical connection suggests the *possible* absence of functional connectivity, while the presence of a functional connection supports the existence of anatomical pathways, which might consist of several segments (edges)^14^. None of the prior work has modeled the neural communication system as a routing problem from an engineering perspective. Routing problems can be formulated using different methods ranging from mixed integer nonlinear programming (MINLP), which are generally NP-Hard, to linear programming (LP) which can be solved efficiently even for problems with millions of variables. Our proposed model is deliberately formulated using LP to ensure it can be solved and scaled to denser networks. The structural-functional brain network model is formulated and solved for each individual brain dataset, unlike prior approaches which generally estimate general mappings between functional and structural connectivity through population analysis. Furthermore, solutions of LP problems implicitly include combinatorial results of the constraints status (active and inactive)^48^, which cannot be characterized by any of the previously proposed models.

The overall brain network estimation is formulated as a network flow problem^49–52^. The capability of conveying information (called *capacity*) is defined by the anatomical strength of pathways, while neural activity at nodes is assumed to be proportional to fMRI activation maps. These maps can be obtained from independent component analysis (ICA), for resting-state fMRI^53,54^, or from statistical activation maps produced via general linear modeling (GLM), for task fMRI^55^. A cost function, minimizing the *information delivery cost* while enhancing the capacity of links most likely to exist, is introduced to define the optimality conditions. With the proposed constraints and cost function, the solution of the network optimization problem defines function-specific circuits using the flow distribution, and improves anatomical connectivity mapping. Our proposed framework focuses on *subject-specific* brain networks, but the model can readily be utilized in population studies.

In Section 2, after detailing the mathematical formulation of the brain network model (Section 2.1), results from brain and phantom datasets are described in Section 2.2 and Section 2.3. Language processing circuits are first identified using dMRI and task-fMRI data from the Human Connectome Project (HCP)^56,57^, and the results are compared with conventional GLM and correlation-based functional networks analysis. Next, results using resting-state fMRI data are presented. Using a diffusion phantom^58–60^, in combination with simulated resting-state fMRI data, a significant improvement in anatomical connectivity mapping is shown. As briefly discussed in Section 3, our results indicate that the proposed model outperforms the GMM method^45^ in finding the maximum number of true connections, with fewer false positives. They also demonstrate the possibility to reliably identify brain circuits associated with specific brain functions. Section 4 provides details about the datasets used in this paper, and the pre-processing steps.

## Results

### Joint structural-functional brain network model

For a network with *N* nodes, *L* = (*N*^2^ − *N*)/2 potential undirected connections exist. Each connection *l* can also be represented as a pair of nodes (*i*, *j*) in order to emphasize the two end points, *i* and *j*. The nodes and edges determine the basic network topology. The notation is summarized in Table 1 and the complete model is presented in Eq. (5) below.

**Table 1:** Definition of Symbols.

Symbol	Description	Type
*i*	Node index: cortical/sub-cortical area	Notation
*l* = (*i*, *j*)	Edge index: pathway connecting node *i* and *j*	Notation
*m*	Functional mode index	Notation
*N*(*i*)	Collection of edges connecting to/from node *i*	Notation
${R}_{i}^{m}$	Functional activation on node *i* associated with functional mode-*m*	Input
*D*_*l*_	Link capacity defined by anatomical connectivity of edge *l*	Input
${f}_{l}^{m}$	Amount of information flow sharing on edge *l* for function *m*	Output
*P*_*l*_	Correction for under-estimated structural connectivity of link *l*	Output
*α*	Unit conversion from capacity to flow	Parameter
*β*	Unit conversion from fMRI activation to flow	Parameter
*γ*	Combined unit conversion $\gamma =\frac{\alpha }{\beta }$ from capacity to fMRI	Parameter
*ρ*	Control favoring/restricting correction for under-estimated connectivity	Parameter

Two fundamental properties can be assigned to the network. For each node *i*, the *average functional activation* ${R}_{i}^{m}$ is obtained from the result of fMRI analysis. The average functional activation approximates the amplitude of local neuronal activity for a specific function (task or resting-state mode). For each link, the *capacity D*_*l*_ is defined by the normalized streamlines count to estimate the strength of the fiber pathway, which models the capability for delivering information. The average functional activation and the capacity associated, respectively, with nodes (cortical/sub-cortical areas) and links (axonal pathways) are the input to the network model.

The network problem consists of two groups of unknown variables: *flow variable*, ${f}_{l}^{m}$, for extracting function-specific circuits and *capacity adjustment variable*, *P*_*l*_, for recovering under-estimated structural connections. The flow variable characterizes the amount of information shared on a link *l* for a given functional mode *m*. The capacity adjustment enables the model to correct for under-estimated structural pathways, and can be added to fulfill the structural-functional constraint. Both variables are unknown a priori and estimated during the model optimization.

**Figure 3:**
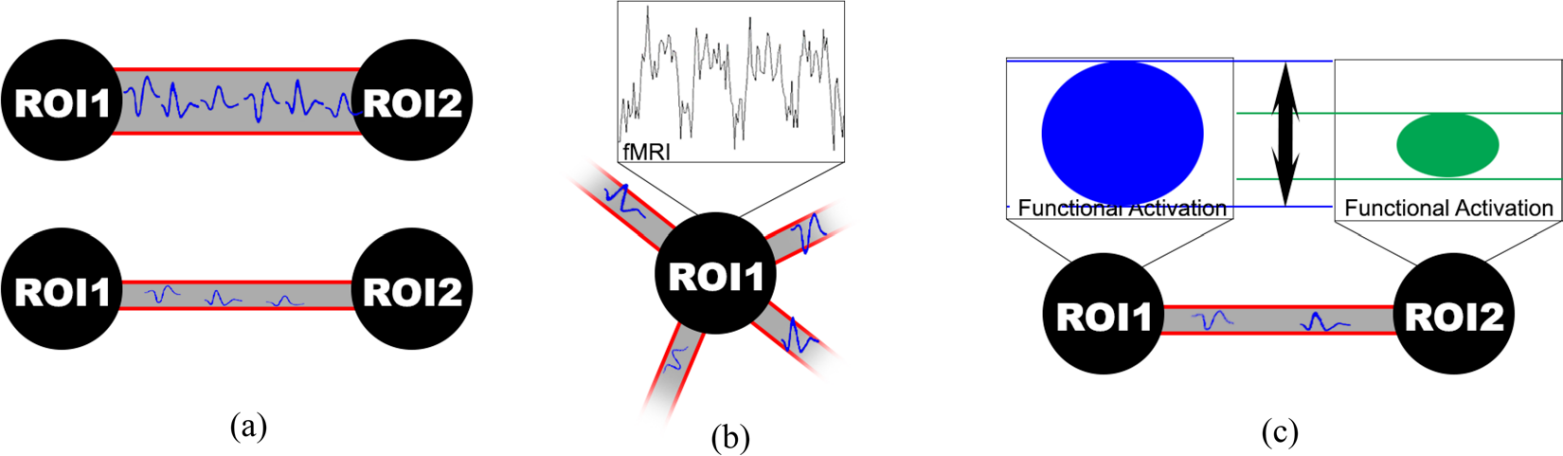
Illustration of constraints enforcing specific relationships between the input data and the unknowns: Panel (a) illustrates the link capacity constraint, formulated in Eq. (1). It indicates that the amount of information which can be delivered through a connection *l* is limited by its anatomical strength (i.e., the connection at the top can deliver more information between ROI1 and ROI2). Panel (b) illustrates the node demand constraint, formulated in Eq. (2). It states that the fMRI signal is the origin/consequence of sufficient amount of information sent/received at a node through all its associated connections. Panel (c) illustrates the feasibility constraint, formulated in Eq. (3). It ensures that information flow is delivered among nodes with sufficient functional activation (i.e., the flow between ROI1 and ROI2 can not exceed the blue functional activation).

In addition to the input data and unknowns, three constraints are proposed to specify and enforce the relationships between the input data and the unknowns. These include: (i) *link capacity constraint*, (ii) the *node demand constraint*, and (iii) the *feasibility constraint*. These are illustrated in Fig. 3, and further described next.

**Constraint 1:** First, the *link capacity constraint* is formulated as

1 $$\sum _{m=1}^{M}{f}_{l}^{m}\le \alpha ({D}_{l}+{P}_{l}),\forall l=1,2,\ldots L.$$

This defines an upper bound on the amount of information which can be carried by a particular connection *l*. In the constraint, the right hand side, *D*_*l*_ + *P*_*l*_, is the total capacity from estimated number of streamlines, and the adjustment for possible under-estimation, while the term $\sum _{m=1}^{M}{f}_{l}^{m}$ is the overall amount of information for all functional modes. This constraint restricts the aggregated flow on link *l*, which cannot exceed its total capacity *D*_*l*_ + *P*_*l*_.

**Constraint 2:** Next, since the signal shared through white matter pathways triggers activity in the associated cortical/sub-cortical areas, the *node demand constraint* is defined as:

2 $$\sum _{l\in N(i)}{f}_{l}^{m}\ge \beta {R}_{i}^{m}\,\forall m=1,2\ldots M,i=1,2,\ldots N.$$

The constraint ensures that the information flow gathered at each node for each individual brain function (e.g., resting-state network) is sufficient to support the brain activity at this node (obtained from fMRI analysis)^47^. In Eq. (2), *N*(*i*) is the collection of all edges connected to node *i*, $\sum _{l\in N(i)}{f}_{l}^{m}$ is the information flow gathered at node *i*, and ${R}_{i}^{m}$ is the average functional activation at this node. Combined with the link capacity constraint, the node demand constraint defines the integration of structural and functional connectivity via the concept of information flow, which unifies brain activity with the underlying structural constraints.

**Constraint 3:** Finally, since the information flow is shared between brain areas, the *feasibility constraint* is defined for any pair of nodes *i* and *j* as

3 $${f}_{l}^{m}\le \beta \,{\rm{\max }}\,\{{R}_{i}^{m},{R}_{j}^{m}\}\,{\rm{where}}\,l=(i,j),\,\forall {l}\,=\,1,2,\ldots L.$$

The constraint guarantees that the flow for a particular connection *l* does not exceed the maximal functional activation ${\rm{\max }}\,\{{R}_{i}^{m},{R}_{j}^{m}\}$ at its two end points *i* and *j*. This additional constraint is needed to make sure that information is delivered and shared between two nodes with at least one node showing an appropriate amount of functional activation. In other words, the constraint prevents arbitrary information sharing between inactive regions.

Finally, we introduce the cost function in Eq. (4). It contains two terms: The first term (left) minimizes the information (flow) delivery cost^27,61–64^. The unit information delivery cost is defined by the reciprocal of the capacity. In other words, connections with high structural connectivity (defined from dMRI data) are associated to lower cost, and preferred by the optimization procedure to transmit information. The second term (right) is used to enhance under-estimated structural connectivity if needed. Since the number of tractography streamlines *D*_*l*_ is related to the probability of existence of a specific white matter pathway, its reciprocal is used as a probabilistic prior in this second term. A constant bias and this probabilistic prior (1/*D*_*l*_) are combined to prevent the unnecessary increase of *P*_*l*_, especially for links with small *D*_*l*_ values.

4 $$\,{\rm{Cost}}\,{\rm{Function}}\,C({f}_{l}^{m},{P}_{l})=\sum _{l=1}^{L}(\frac{1}{{D}_{l}}\sum _{m=1}^{M}{f}_{l}^{m})+\rho \sum _{l=1}^{L}[(1+\frac{1}{{D}_{l}}){P}_{l}]$$

After summarizing all the constraints, cost function, input data and variables, combining the unit conversion parameters to be $\gamma =\frac{\alpha }{\beta }$, and incorporating *β* into the definition of ${f}_{l}^{m}$ (from now on ${f}_{l}^{m}=(original\,{f}_{l}^{m})/\beta$), the overall network optimization problem is summarized as:

5 $$\begin{array}{ll} & \mathop{{\rm{\min }}}\limits_{{f}_{l}^{m}\ge 0,{P}_{l}\ge 0}\sum _{l=1}^{L}(\frac{1}{{D}_{l}}\sum _{m=1}^{M}{f}_{l}^{m})+\rho \sum _{l=1}^{L}[(1+\frac{1}{{D}_{l}}){P}_{l}]\\  & {\rm{subject}}\,\mathrm{to}:\\  & \sum _{m=1}^{M}{f}_{l}^{m}\le \gamma ({D}_{l}+{P}_{l}),\forall l=1,2,\ldots L\\  & \sum _{l\in N(i)}{f}_{l}^{m}\ge {R}_{i}^{m}\,\forall m=1,2\ldots M,i=1,2,\ldots N\\  & {f}_{l}^{m}\le \,{\rm{\max }}\,\{{R}_{i}^{m},{R}_{j}^{m}\}\,{\rm{where}}\,l=(i,j),\forall l=1,2,\ldots L\end{array}$$

In practice, the true value of the unit conversion parameter *γ* is not known, but it is an important parameter which determines the feasibility of the problem. A too small *γ* may eliminate all the possible solutions and lead to an empty feasible set. On the other hand, a too large *γ* may introduce many arbitrary solutions. Therefore, to ensure feasibility, *γ* was determined as the largest ratio (over all nodes) between the total functional activation and total structural connectivity, as defined below

$$\gamma =\mathop{{\rm{\max }}}\limits_{i}\frac{\sum _{m}{R}_{i}^{m}}{\sum _{l\in N(i)}{D}_{l}}$$

The proposed joint structural-functional network model is a linear programming problem. This can be done efficiently for even millions of variables with the interior point method^65^. In this paper, a general linear programming solver, available in the IBM ILOG CPLEX Optimization Studio [https://www-01.ibm.com/software/commerce/optimization/cplex-optimizer/], is utilized.

Next, results from brain (HCP) and phantom (Fibercup) datasets are presented to demonstrate the capability of the proposed method to identify function-specific brain circuits, and to enhance structural connectivity estimation. In particular, we focus on the HCP task fMRI data targeting language processing areas (e.g., inferior frontal, superior temporal, anterior cingulate cortex) through the auditory presentation of mathematical problems and comprehension questions from stories^66^. We also use the HCP resting-state data, and compare all our results to the literature and existing neuroanatomical knowledge. Additionally, we use the realistic Fibercup diffusion phantom, combined with synthesized resting-state fMRI signal, to illustrate how the proposed model can recover missing or under-estimated connections. Because ground truth structural connectivity is available for the Fibercup phantom, we decide to focus on this dataset to assess the performance of the method to enhance structural connectivity mapping. Additionally, we compare our model to existing state-of-the-art methods and assess its sensitivity to variations in fMRI data quality.

### Discovering function-specific anatomical circuits using the Human Connectome Project data

Twenty-five healthy subjects were randomly selected from the HCP ConnectomeDB database [https://db.humanconnectome.org/], to demonstrate the extraction of language processing circuits and resting-state networks^67^. Results for the proposed joint structural-functional connectivity model are presented, and compared with structural and functional (using Pearson correlation) networks.

#### Language processing circuits

Without functional information, whole brain anatomical networks, estimated from dMRI using deterministic tractography, are largely dominated by the main white matter pathways (e.g., corpus callosum, superior longitudinal fasciculus). For reference, we illustrate this effect in Fig. S1 (Supplementary Material), which shows the consistency (across subjects) of the “strongest” structural connections. It can be noted that the top 20 links are consistently located between sub-cortical areas, as well as subcortico-cortical pathways.

**Figure 4:**
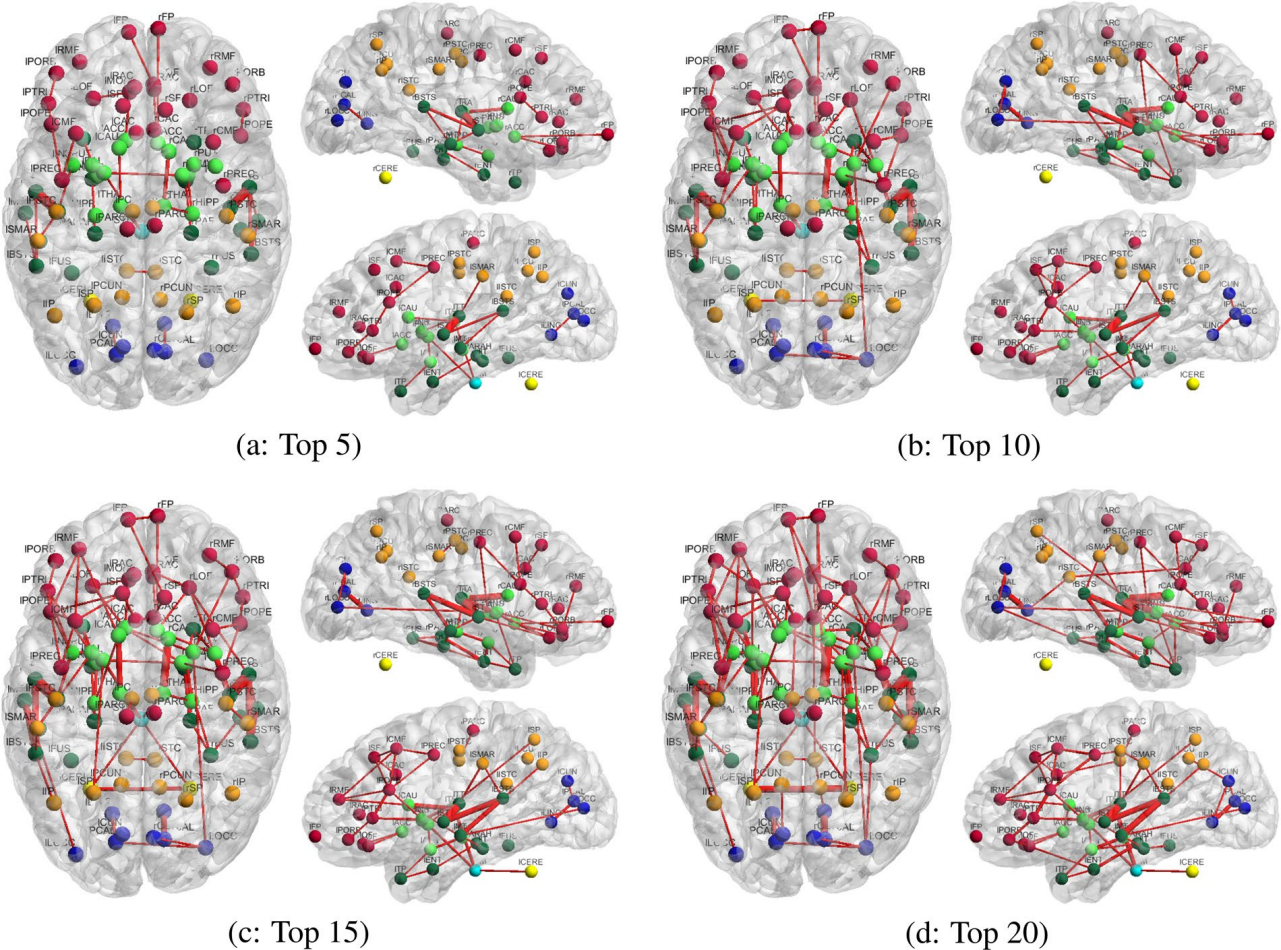
Consistency of the top (strongest) structural-functional connections of the language processing areas, estimated using the proposed information flow model is shown in panels a (top 5), b (top 10), c (top 15) and d (top 20). The graphs are generated by selecting the top 5, 10, 15 and 20 connections from each individual subject. The edges’ width, for each connection, represents the consistency across subjects, i.e., the frequency of identification across all subjects for a given threshold (5, 10, 15, 20): The thicker the edge is, the more consistently the link is identified as a top-ranked connection across individuals. The nodes color represents the anatomical location: red for frontal, orange for parietal, blue for occipital, green for temporal, bright green for sub-cortical, yellow for cerebellum and bright blue for brainstem. Abbreviations for region labels are provided in Table S1 (Supplementary Material).

Figure 4 shows the consistency (across the twenty-five subjects) of the identified top-ranked connections using the structural-functional (flow) brain network model. For comparison, results from the conventional functional connectivity network are also presented in Fig. S2 (Supplementary Material).

The functional connectivity network (Fig. S2, Supplementary Material) is calculated by first running, for each subject, a general linear model (GLM) analysis contrasting the comprehension questions and math problems with baseline. The maps of regression parameter estimates were combined to identify brain areas activated by either one of the tasks. This step localizes language processing areas such as the inferior lateral frontal cortex, and temporal cortex. We chose to use this approach, rather than contrasting the comprehension questions versus math problems^66,67^, to attempt to recover more areas, involved in arithmetic tasks, which have been shown^68^ to recruit brain networks overlapping with language comprehension areas (e.g., inferior frontal gyrus). Subsequently, for all possible pairs of those areas, the Pearson correlation coefficient between their respective fMRI signal is computed. It is used to define the weight of the corresponding edge in the (functional) brain network.

In Fig. 4, brain connections which are consistently identified across subjects by the proposed method are mostly located in the temporal and pre-frontal cortices, with slightly stronger connectivity or more connections identified in the left hemisphere, as expected from known lateralization of the language functions in humans. In addition, some connections are also identified with the occipital, parietal cortex, and sub-cortical areas.

In particular, as shown in Fig. 4(a), pathways from/to the superior temporal cortex, bilaterally (rST and lST), where the primary auditory cortex is located, are identified as the most consistent across subjects, and most significant (ranked as top 5) by the proposed approach. Specifically, we find that these pathways include connections between: the superior temporal and middle temporal cortices, the superior temporal cortex and supramarginal gyrus (Brodmann area 40, involved in reading), and the superior temporal cortex, transverse temporal cortex, insular cortex, pars opercularis (Broadmann area 44, involved in semantic tasks), pars triangularis (Broadmann area 45, also involved in semantic tasks), and the precentral gyrus.

Those pathways were reported in prior work on the human language and speech processing pathways^69–73^ as connections between the primary auditory cortex, Wernicke’s area (posterior part of the superior temporal gyrus) and Broca’s area (Brodmann areas 44 and 45), and from the primary auditory cortex to the supplementary motor areas.

In contrast, the correlation-based functional networks shown in Fig. S2 (Supplementary Material) identify a large number of inter-hemispheric connections between language processing areas, as a result of similarity between the fMRI time-courses from homologous brain areas. It can be observed that the functional networks connect many areas, that are potentially related to the auditory language task. Many of those direct functional connections do not necessarily reflect direct structural connectivity (e.g., between the left and right banks of the superior temporal sulci, or the left and right transverse temporal cortices) and it is therefore not possible to infer information about the underlying structural circuits. We note that our proposed approach identifies circuits with patterns which are similar to recent results reported in the literature^69^. Moreover, our joint structural-functional network model identifies connections with greater consistency across subjects (i.e., edge thickness in Figs S1, S2 and 4), by comparison with the functional or structural networks.

**Figure 5:**
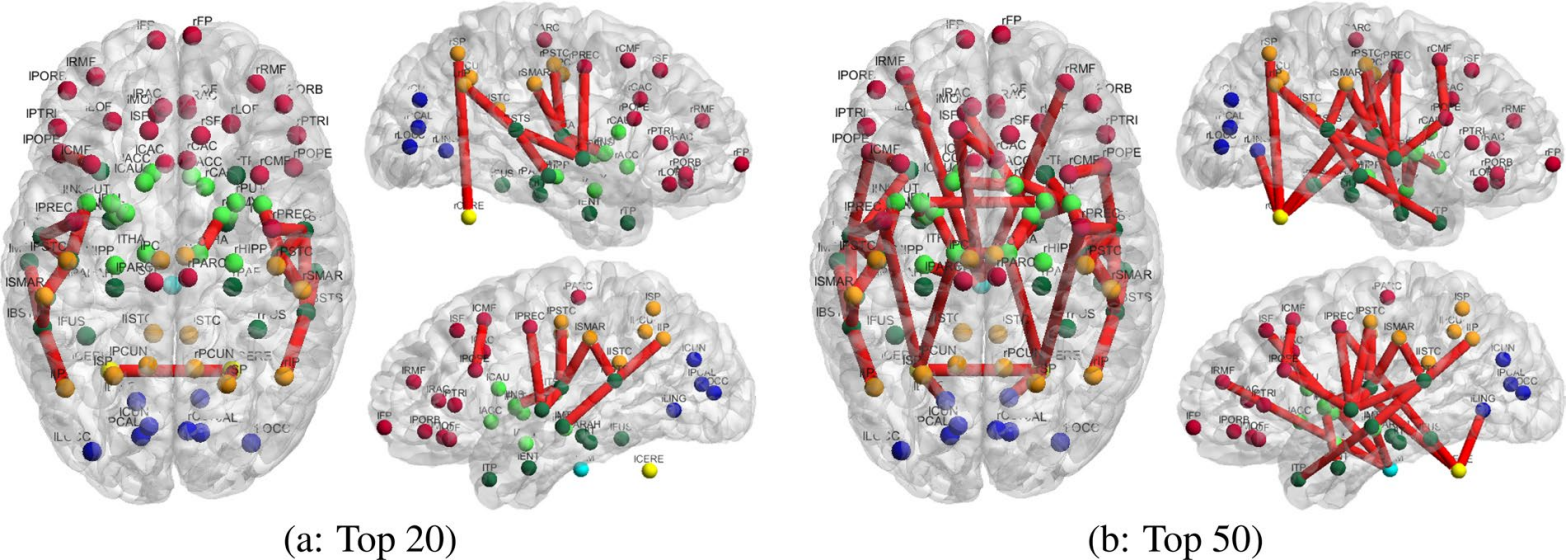
Top 20 (**a**) and 50 (**b**) structural-functional connections of the language processing areas, with the strongest mean normalized connectivity (across subjects). Networks are estimated using the proposed information flow model. Edges’ width is proportional to mean connectivity value across subjects. The color code and labels are identical to Fig. 4.

In addition to results on the consistency of connections, networks representing the top 20 or 50 connections, with the highest average “strength” (across subjects), are presented in Figs 5, S3 and S4 (Supplementary Material), respectively for joint structural-functional (flow), structural, and functional connectivity. In these figures, the edges’ thickness is proportional to the average connectivity values. Additionally, the complete lists of connections corresponding to the 50 pathways with strongest connectivity values are presented in Supplementary Material Tables S2, S3 and S4.

To further characterize the full distribution of connectivity values (over all edges of the network), scatter plots of their mean (across subjects) and standard deviation are presented in Fig. S6 (Supplementary Material) for structural, functional and joint structural-functional connectivity, respectively. Contrary to Figs 4, 5, S1, S2, S3, and S4, the scatter plots in Fig. S6 do not rely on the selection of edges with the highest connectivity (e.g. top 5), and therefore provide complementary information about the range of connectivity values, and their variability. Each point (blue or red) in the scatter plots represents a connection between a specific pair of brain areas. The red points correspond to connections with at least one node in the temporal cortex (language processing areas). For example, the cluster of red points with mean value close to 1 and standard deviation close to 0, in the scatter plot for the joint structural-functional network, corresponds to the connections in Fig. 5 (Panel a) located in the temporal and parietal areas.

**Summary:** Figs 4, 5, S1, S2, S3, S4, and S6 (Supplementary Material), and Tables S2, S3 and S4 (Supplementary Material) illustrate some of the advantages of our proposed joint structural-functional (flow) brain network model to leverage functional connectivity data in order to identify function-specific brain circuits, and simultaneously enhance corresponding pathways. In particular, we show that our approach successfully isolates twenty-four pathways specific to language processing functions, all of them having at least one node located in the (left or right) temporal lobe (see Fig. 5(b)). Moreover, we find that the vast majority of these connections are located within each hemisphere, while few inter-hemispheric pathways were found through the corpus callosum^74^.

These connections, including pathways between the superior temporal and transverse temporal sulci, transverse temporal sulcus and insula, transverse temporal sulcus and postcentral gyrus, transverse temporal sulcus and supramarginal gyrus, banks of the superior temporal sulcus and middle temporal sulcus, precentral gyrus and superior temporal sulcus, belong to the language processing circuit^69–73^. In contrast, the (non function-specific) structural network only identified eight connections related to language processing.

For the functional connectivity, nineteen connections associated with areas located in the temporal lobes were identified, with the majority (eleven) located between inter-hemispheric locations. These reflect strong functional correlation between cortical areas with similar response to the stimulus used in the task fMRI experiments, rather than language processing circuits. Since our proposed method jointly leverages functional and structural connectivity information, it exhibits the closest pattern to the known structural connectivity basis of language processing^69–74^.

#### Resting-state circuits

**Figure 6:**
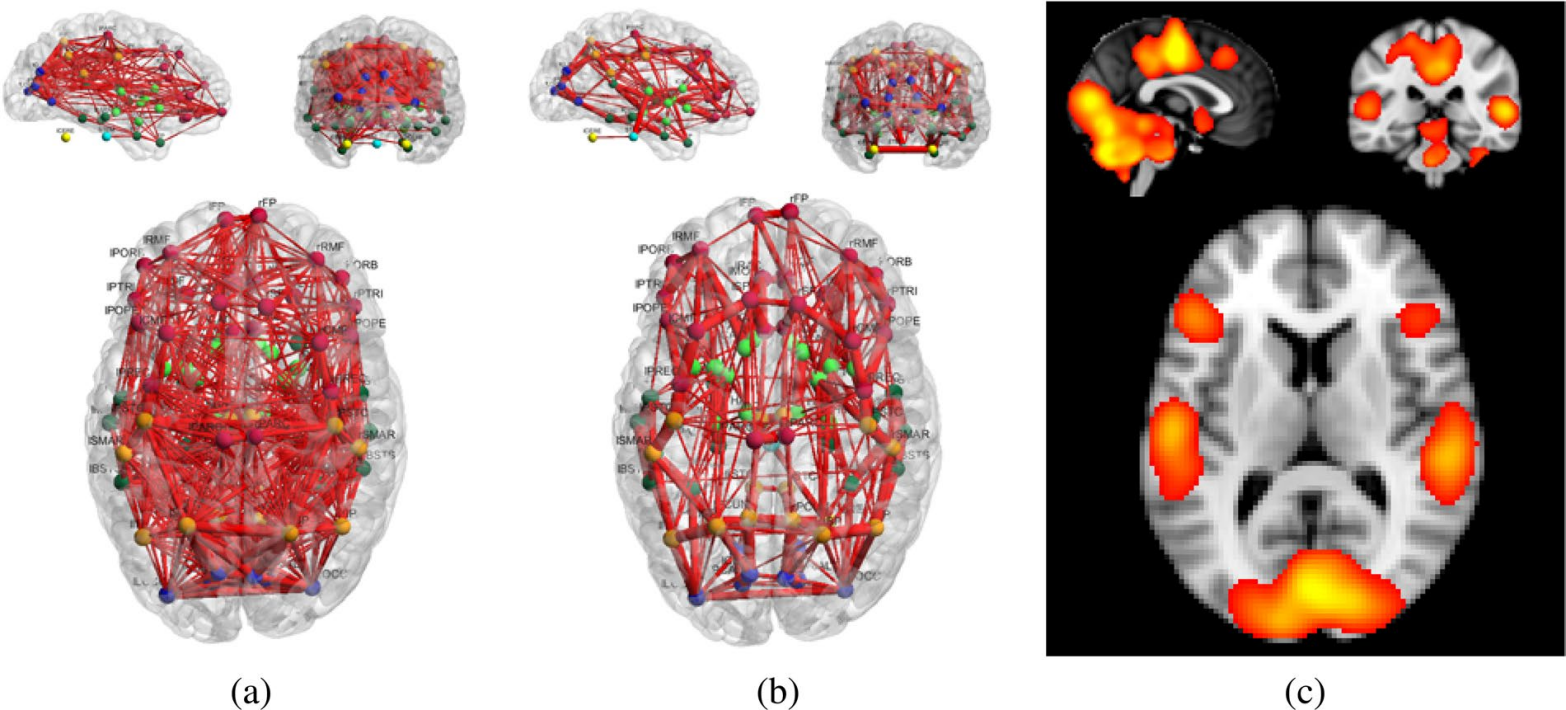
Resting-State Circuits estimated from the 25 HCP subjects using correlation-based functional connectivity (a) and the proposed joint structural-functional model (b). The structural connectivity information depicted in Fig. S1 (Supplementary Material) was used to generate results in panel (b). Additionally, an aggregate of the resting-state activation maps, obtained from ICA analysis of data from the BrainMap database^37^, is shown in panel (c) as a reference. In panels (a) and (b), the edges’ width represents the frequency of occurrence across subjects, for the 100 strongest connections. In panel (b), the network shown is an aggregate of all brain circuits corresponding to the 10 ICA components (i.e., ICA *modes*) shown in Fig. 7.

In this section, we utilize resting-state fMRI^75^, in combination with dMRI data, to identify brain circuits associated with specific brain functions (e.g., motor, audition, vision). The overall resting-state (correlation) functional network and joint structural-functional (flow) network are presented in Fig. 6. In addition, and as a reference, we show an average resting-state activation map, obtained from the ten most reliable components from the 20-dimensional ICA analysis [https://www.fmrib.ox.ac.uk/datasets/brainmap+rsns/] of the BrainMap database^37,76^. In this section, functional connectivity is estimated by the Pearson correlation of resting-state fMRI time courses between cortical/sub-cortical areas, and the average values of the ICA spatial maps within each cortical/sub-cortical area define the ${R}_{i}^{m}$ for the joint structural-functional network model (See Section 4).

The networks in Fig. 6(a) and (b) illustrate the frequency of occurrence, across all subjects, of each connection (represented as the edges’ width), after selection of the 100 strongest connections for each subject. Both functional and joint structural-functional (flow) networks contain nodes with a high number of connections, which are in agreement with activations shown on the map in panel (c). However, it can be noted, as for the language processing circuits analysis, that the functional network presents a large number of connections between brain areas likely to not be anatomically connected. The proposed joint model generates an overall structural-functional network which is in agreement with recent works on the interaction between structural and functional networks^13,77^.

**Figure 7:**
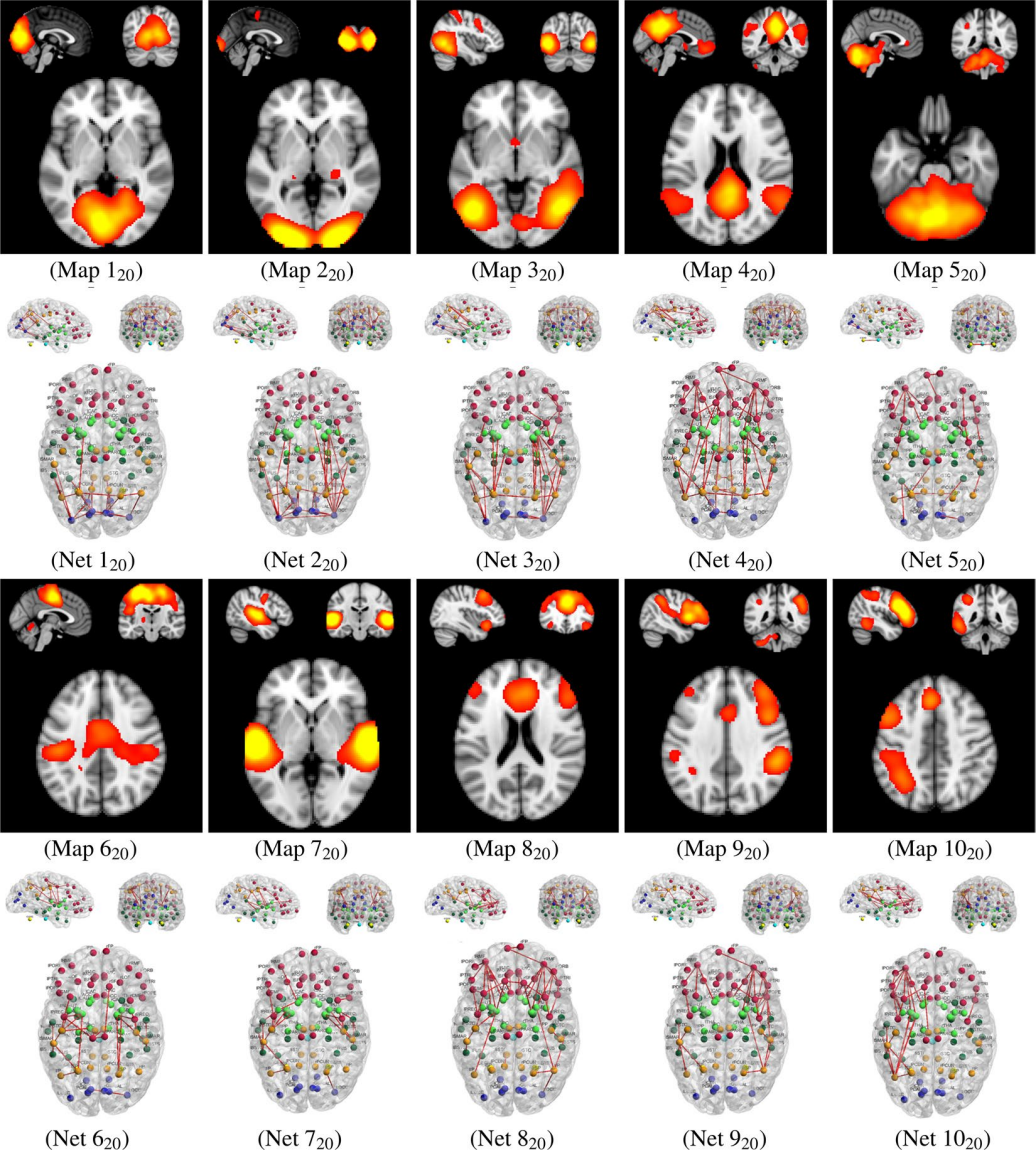
Resting-state structural-functional networks (e.g., Net 1_20_), for the ten most reliably identified resting-state spatial maps (e.g., Map 1_20_) using a 20-dimensional ICA decomposition of the BrainMap data^37^: Each map and network correspond to a major brain function (following the naming convention from Smith *et al*.^37^). We show results for the visual network (Maps and Nets 1_20_, 2_20_ and 3_20_), default mode network (Map and Net 4_20_), cerebellum network (Map and Net 5_20_), sensorimotor network (Map and Net 6_20_), auditory network (Map and Net 7_20_), executive control network (Map and Net 8_20_) and frontoparietal network (Maps and Nets 9_20_ and 10_20_). All spatial maps (*i*_20_) were converted to *z* statistics using a mixture-model fit and thresholded between *Z* = 1.5 and *Z* = 5, and the structural-functional networks represent the corresponding brain circuits generated by our proposed method. The edges’ width represents the frequency of occurrence of the strongest connections across the 25 subjects.

Following the naming convention for the ten most reliably identified ICA spatial maps^37^, we present results from the joint structural-functional network model in Fig. 7. We demonstrate the capability of the proposed model to identify brain circuits associated with specific brain functions. Each of the ten primary ICA maps corresponds to a major brain function, such as the auditory or motor network, and we therefore aim to identify the corresponding structural sub-networks. By incorporating functional and structural information, we further aim to enhance the identification of those structural connections (as compared to, e.g., pure tractography results with initialization in activated areas). Well-known brain circuits^13,16,78–80^ are consistently identified across subjects, using our proposed joint network model. Figure 7 provides a visual overview of the findings, which are summarized next. We recall that, for example, Map 1_20_ denotes spatial component number 1 from the 20-dimensional ICA decomposition of the BrainMap data^37^, and Net 1_20_ denotes our corresponding structural-functional network, estimated using the proposed joint (flow) network model for the twenty-five HCP subjects. The ICA modes from our analysis of the HCP datasets were identified by matching them to the ten spatial components from the BrainMap data, using correlation.

- **Visual Network (Maps and Nets** 1_20_, 2_20_, 3_20_**)**: Homologous areas of the occipital and parietal cortices were found to be connected, via the corpus callosum^16^. In Net 1_20_, the cuneus, pericalcarine cortex, lateral occipital cortex and inferior/superior parietal cortices were consistently identified as part of this inter-hemispheric circuit, with a certain emphasis on the medial visual areas. In addition, we found the lingual gyrus to be connected with the pericalcarine cortex, within each hemisphere, and so was the isthmus of the cingulate gyrus with the lingual gyrus and the precuneus (medial part of Brodmann area 7, involved in vision and proprioception)^81^. Consistent intra-hemispheric connections between the precuneus and cuneus were also identified. Finally, a strong and lateralized connection (left hemisphere only) between the inferior parietal cortex and the banks of the superior temporal sulcus^82^ was found.
  In Net 2_20_, greater connectivity with the occipital poles was found. Bilaterally, the lateral occipital pole was found to be connected with the fusiform gyrus (involved in visual recognition) and temporal cortex. We also identified connections, within each hemisphere, between the superior parietal cortex and thalamus.
  In Net 3_20_, similar connectivity patterns were observed with lateral pathways dominating. The superior parietal lobule (involved in visuospatial orientation and receiving visual input) was notably connected with the thalamus, insula and pallidum.
- **Default Mode Network (Map and Net** 4_20_**)**: Consistent with the literature on cortical/sub-cortical areas involved in the default mode network^83–86^, the joint structural-functional network model identified connections between the medial parietal cortex, bilateral lateral parietal cortex and frontal cortex. In particular, the cingulum tract plays an important role^36,84^ in the connection of the precuneus and posterior cingulate cortex with the medial frontal cortex. Bilateral connections between the cuneus, precuneus, isthmus of the cingulate cortex, posterior cingulate cortex, rostral/caudal anterior cingulate cortex and frontal cortex (including the rostral frontal cortex, superior frontal cortex, medial orbitofrontal cortex, and pars opercularis/triangularis/orbitalis) were consistently identified across the twenty-five subjects. In addition, connections between the medial parietal cortex and the inferior/superior parietal cortex and supramarginal gyrus were found.
- **Cerebellum Network (Map and Net** 5_20_**)**: Because the cortical/sub-cortical labeling used in this work (see Section 4) creates only two labels for the left and right parts of the cerebellum, identification of connections between specific lobes or lobules was not possible. Nonetheless, connections between the left and right cerebellum were consistently found with the brainstem (as well as with each other), with additional connections to the thalamus and frontal/occipital cortex.
- **Sensorimotor Network (Map and Net** 6_20_**)**: We found consistent connections (across subjects), via the corpus callosum, between the left and right paracentral lobules (which include the supplementary motor area and somatosensory functions). Within each hemisphere, we found the paracentral lobule to be connected to the thalamus, via the posterior cingulate cortex, and to the precentral cortex. Consistent connections were also found bilaterally between the postcentral cortex, the supramarginal gyrus, banks of the superior temporal sulcus and superior temporal cortex.
- **Auditory Network (Map and Net** 7_20_**)**: The resting-state auditory network is known to include the superior temporal gyrus, Heschl’s gyrus and the posterior insular cortex. Using the joint structural-functional network model, we identified circuits within each hemisphere (no inter-hemispheric connections were found), with greater connectivity in the left hemisphere. This increased connectivity may reflect known lateralization of the language areas in humans. Bilateral connections between the superior temporal sulcus and several other areas were found, which include: the middle temporal gyrus (involved in reading), banks of the superior temporal gyrus (involved in voice interpretation) and transverse temporal gyrus (Heschl’s gyrus). The superior temporal gyrus was also found to be connected to the insula.
- **Executive Control Network (Map and Net** 8_20_**)**: Connections for the executive control network were essentially located within the frontal areas. The rostral anterior cingulate was found to be connected, bilaterally, with the superior frontal cortex, caudal anterior cingulate, and nucleus accumbens. The rostral middle frontal cortex was also found to be heavily connected to other frontal and sub-cortical areas, including the caudal middle frontal cortex, pars orbitalis, caudate nucleus, pallidum, nucleus accumbens, and insula. Interestingly, putative connections between the frontal areas and the inferior parietal cortex (via the striatum) were identified too, and may be part of the frontoparietal system^87^ which is associated with attention and the detection of novel events.
- **Frontoparietal Network (Maps and Nets** 9_20_, 10_20_**)**: Both networks 9_20_ and 10_20_ were found to be highly lateralized and mirrored. In network 9_20_, several frontal areas (with the rostral middle frontal cortex and pars triangularis playing a central role) were identified as connected to parietal areas via the insula, putamen and pallidum. In network 10_20_, similar frontal areas were identified as connected to parietal and temporal (language) areas via the insula, putamen and pallidum.

Those results illustrate the ability of the proposed method to reliably identify function-specific brain circuits, and better capture the structural connectivity pattern supporting these functions. This opens potential new avenues to investigate changes in those networks, for instance in the context of development/aging and neurodegeneration.

### Enhanced structural connectivity mapping and validation using a phantom dataset

In order to demonstrate the ability of the proposed model to recover structural connections that are typically under-estimated when only using dMRI tractography, we focus next on a phantom dataset [http://www.tractometer.org/original_fibercup/data/] (Fibercup)^58–60^. These results are presented for validation and performance evaluation, which would be difficult to achieve without ground truth information. Despite their relatively simple nature, these experiments are meant to confirm the ability of our model to enhance structural connectivity mapping and further support results presented in Section 2.2. First, we present results using structural and functional connectivity information separately to illustrate the possible shortcomings and limitations of such approaches^25^. Second, the performance of the proposed joint model is examined by comparison with the ground truth connectivity information available for the phantom data. Finally, the performance of our proposed method is compared with the closest joint modeling method^45^ to our work.

#### Separate structural and functional connectivity analyses

**Figure 8:**
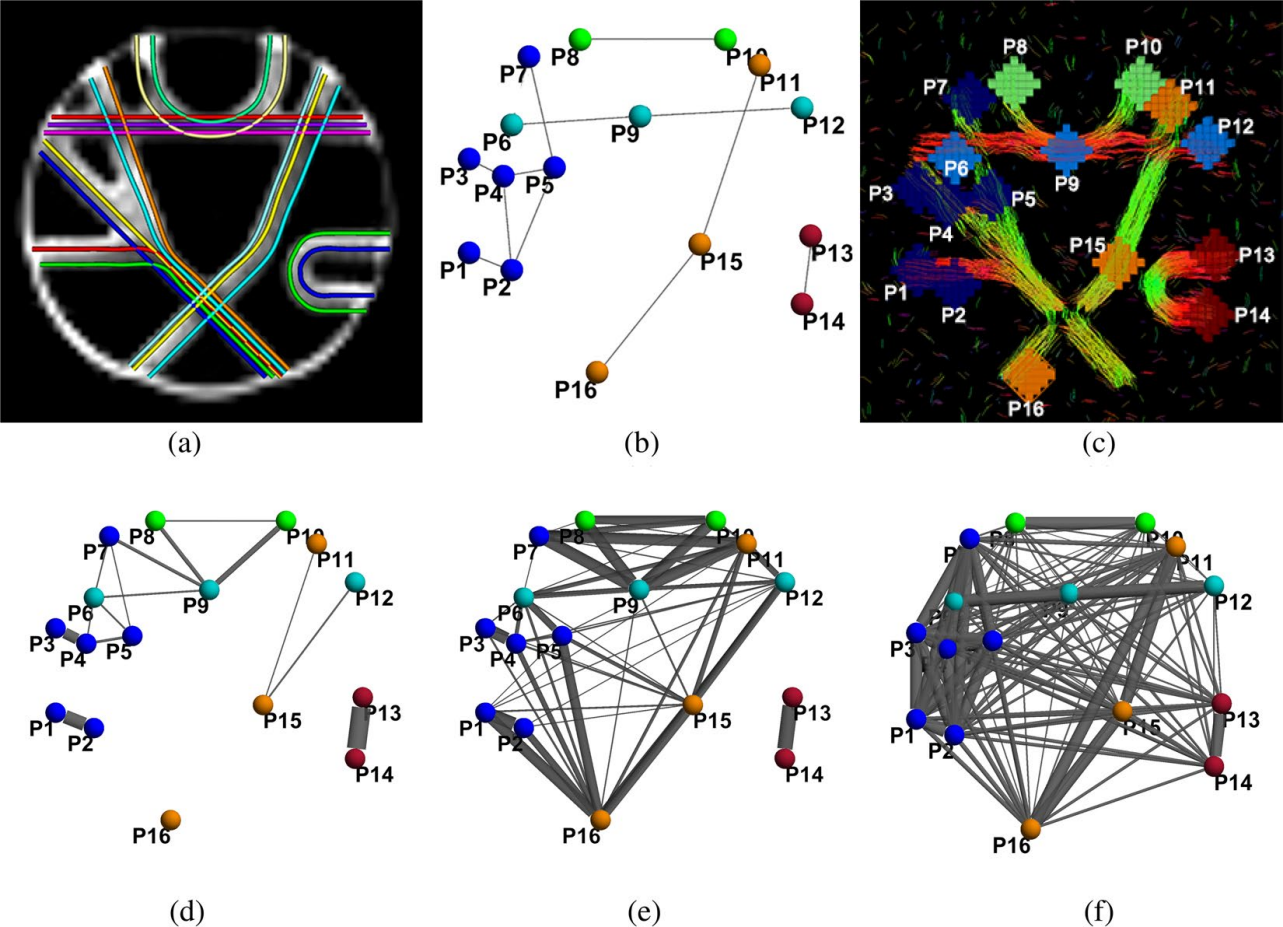
Fibercup^58–60^ structural and simulated functional connectivity: Panel (a) shows the ground truth fibers and panel (b) shows the corresponding ground truth structural network, with endpoints (nodes) Px sharing the same color if they belong to the same sub-network. Panel (c) illustrates results obtained using deterministic tractography and panel (d) shows the corresponding structural network. Panel (e) shows another structural network, obtained from probabilistic tractography. Panel (f) shows the functional network, obtained from simulated fMRI data (see text and Fig. S5 in Supplementary Material). The edges’ width represents streamlines count in panels (d and e), and magnitude of fMRI correlation in panel (f). Panel (a) is adapted from Fig. 4 in Fillard *et al*.^58^.

Here, we describe results for connectivity networks using deterministic and probabilistic tractography methods, as well as functional correlation from simulated fMRI signals, as shown in Fig. 8. The functional network was obtained from the average (over 100 realizations) correlation between fMRI signals simulated at each of the sixteen end-points (P1, …, P16), using the approach described in Section 4.

In Fig. 8, panels (c) and (d) highlight some of the typical limitations of deterministic tractography approaches. In areas where fibers cross or diverge, streamlines stop or may follow erroneous directions. This results in both false-positive (spurious) and false-negative (missed) pathways (e.g., connections P7-P9, P8-P9 and P15-P16). In general, deterministic tractography appears to be more prone to false-negative (missing) connections. Conversely, probabilistic tractography, because of its ability to explore a larger set of candidates streamlines, can be prone to false-positive (spurious) connections. In Fig. 8(e), although few connections are missed, it can be observed that several spurious pathways were indeed identified.

Functional MRI data was generated via realistic simulations, using the SimTB Toolbox^88^, so that nodes belonging to the same sub-network share a common activity pattern (see Section 4). Similar to brain data, the correlation-based functional network, illustrated in Fig. 8(f), successfully captures the sub-networks (identified by the wider edges). However, within a given sub-network, all nodes are strongly connected and it is therefore impossible to extract the underlying structural circuits. Moreover, partial correlations across sub-networks (e.g., between the red and blue sub-networks) can also make it challenging to identify those circuits.

We now illustrate how the joint modeling of structural and functional connectivity information can help identify the five (blue, green, orange, cyan, red) “function-specific” circuits, while also improving the reconstruction of under-estimated structural pathways.

#### Joint structural-functional connectivity analysis

We assessed the robustness of our model to variability and imperfections in the fMRI data. 100 random instances of the fMRI signals were created as described in Section 4, by modulating the noise level and amplitude of the signal at each voxel within the end points (P1, …, P16). Figure S5 (Supplementary Material) illustrates how a standard ICA analysis (similar to the results presented in Section 2.2) leads to “resting-state” networks which coincide with the five networks shown in Fig. 8(b). We use these maps to quantify the functional correlation between nodes. Because of noise, it can be noted that certain maps in Fig. S5 (Supplementary Material) (e.g., panels (a), (b), (f) and (g)) include weak functional correlation with incorrect modes. In panel (b), for instance, the blue sub-network is clearly identified, as well as moderate connectivity with the red network (which should ideally only appear in panel (e)). This is intended and designed in the fMRI simulation method to test the robustness of the proposed model to imperfect functional data.

**Figure 9:**
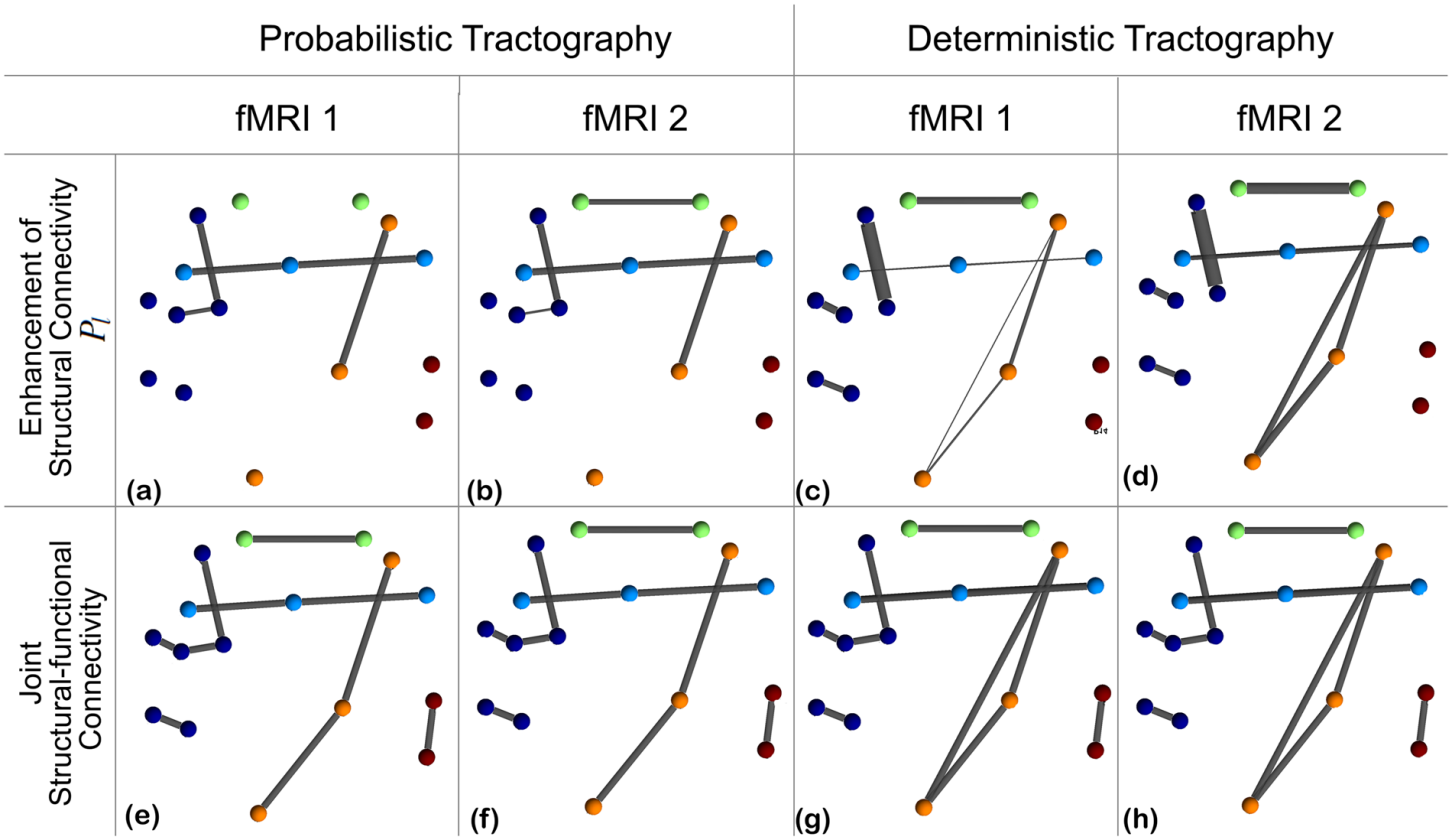
Circuits estimated using the proposed joint structural-functional network model with two different realizations of the fMRI signal (fMRI 1: top row from Fig. S5; fMRI 2: bottom row from Fig. S5 in Supplementary Material). Despite the variation in the fMRI data, results are very similar for each tractography method. Networks in the top row illustrate the incremental connectivity for connections that are under-estimated by tractography analysis only (e.g., P5-P7). Networks in the bottom row demonstrate the joint networks obtained from the proposed scheme, that correctly estimates nearly all connections from the ground truth data.

In Fig. 9, we present the results for the proposed joint structural-functional network model, combining structural connectivity from probabilistic (left) or deterministic (right) tractography with fMRI data. Two representative but different realizations of the simulated fMRI data were used (fMRI 1 and 2), as illustrated in Fig. S5.

We focus first on the enhancement of structural connectivity (top row) and emphasize that, since structural connectivity values are different between deterministic and probabilistic tractography, it is not meaningful to directly compare link widths. However, we note that enhancement of (under-estimated) structural connectivity (i.e., parameter *P*_*l*_ in Table 1) occurs more often in the deterministic tractography case (links P1-P2, P3-P4, P8-P10, P15-P16), which is possibly due to the fact that deterministic tractography tends to “miss” more connections. In addition, most links involved in “fiber crossing” configurations, and therefore more susceptible to being under-estimated, (e.g., P5-P7, P6-P9, P9-P12, and P11-P15) were enhanced in all cases. Finally, a spurious “direct” connection between P11 and P16 is created by the algorithm. We hypothesize that this is a consequence of the connection P15-P16 being missed by the deterministic tractography method, which leads to equal probability of existence for links P11-P16 and P15-P16. The joint structural-functional connectivity network successfully recovers nearly all connections present in the ground truth network.

**Figure 10:**
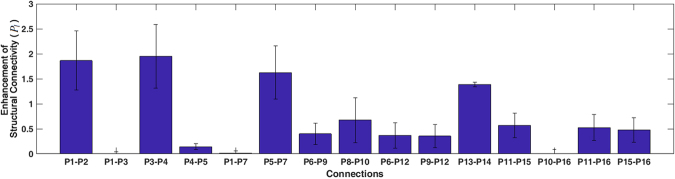
Enhancement of structural connectivity: Mean and standard deviation of the correction parameter (*P*_*l*_ in Table 1) for under-estimated structural connectivity of each link. These results were generated using deterministic tractography and 100 simulated fMRI instances. Connections P5-P7, P6-P9, P8-P10, P9-P12, P11-P15 and P15-P16 are consistently enhanced.

Additional experiments were performed, for deterministic tractography and with 100 different functional connectivity networks, to assess the reproducibility of results from Fig. 9. Figure 10 provides the mean and standard deviation, for each connection, of the correction parameter *P*_*l*_ for under-estimated structural connectivity. This graph demonstrates that the proposed method consistently recovers problematic connections, including pathways with crossing or bending configuration, such as P5-P7, P6-P9, P8-P10, P9-P12, P11-P15 and P15-P16. In particular, P6-P9, P8-P10 and P11-P15 are not well reconstructed by deterministic tractography and therefore benefit from this approach. Finally, false-positive connections obtained from tractography, such as P6-P7, P7-P9, P8-P9, P9-P10, and P12-P15 are never enhanced (*P*_*l*_ = 0).

**Figure 11:**
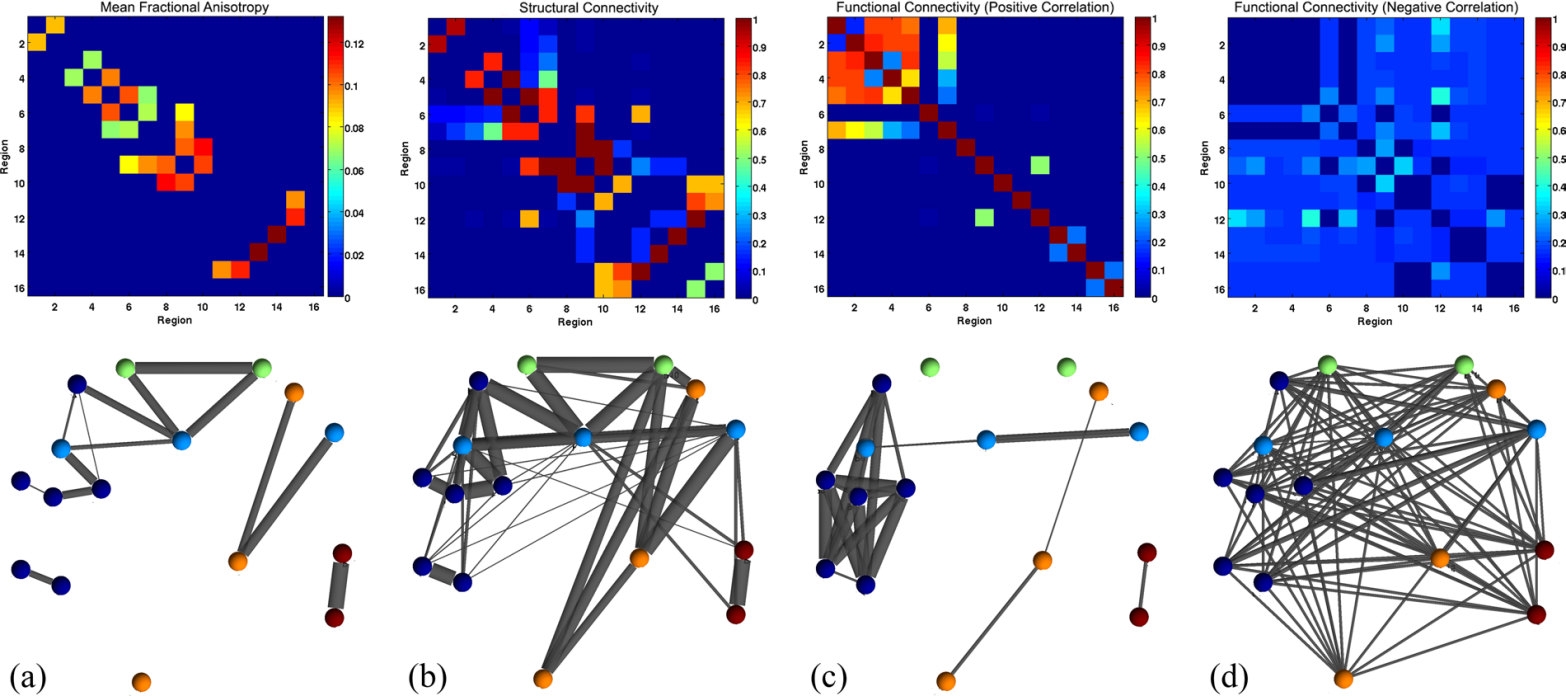
Comparison with the Gaussian Mixture Modeling (GMM) approach by Venkataraman *et al*.^45^: In this joint probabilistic model, the mean fractional anisotropy (FA) along connections (Panel a) is used as an estimate of structural connectivity, and combined with (correlation) functional connectivity (as shown in Fig. 8(f)). The probabilistic model, using the EM algorithm, determines whether each potential link is anatomically connected (Panel b) and functionally positively (Panel c) or negatively (Panel d) correlated. The top row shows connectivity matrices, while the bottom row shows corresponding network representations.

Lastly, we compare our proposed method with a recent joint probabilistic framework based on Gaussian Mixture Modeling (GMM)^45^, which is also the closest work to ours. In this approach, structural connectivity (defined by the mean fractional anisotropy along pathways) and functional connectivity (correlation) are assumed to be drawn from one of six joint Gaussian distributions, which model the probability of being anatomically connected, functionally positively correlated, and functionally negatively correlated. The parameters for the joint GMM are learned using the expectation maximization (EM) algorithm, and the maximal posterior estimator is used to determine which of the six categories each link belongs to. The mean FA of the Fibercup phantom is shown in Fig. 11(a). The mean FA indicates that the links P1–P2, P4–P5, P5–P6, P6–P9, P7–P9, P8–P9, P8–P10, P9–P10, P15–P11, P15–P12, and P13–P14 are connected. Among them, five links correspond to false positives and four links are missing. Figure 11(b) shows how frequently each structural connection is identified, over 100 trials. The model is able to correctly recover the four missing connections P3–P4, P5–P7, P9–P12 and P16–P15. However, it also generates several other spurious connections. Results for positively/negatively correlated functional networks are shown in Fig. 11(c) and (d), respectively. In panel (c), we note that the connection between nodes P8 and P10 is missing, and that spurious connections between P1, P2, P3, P4, P5 and P7 are generated. Based on these results, we conclude that our proposed model produces improved results, as it minimizes the number of false positive connections while still recovering most ground truth connections (see Fig. 9 compared to Fig. 11(b)).

## Discussion

We have introduced a joint structural-functional brain network model using the concept of information flow, which integrates diffusion MRI and functional MRI data to enable the identification of function-specific anatomical circuits from whole brain networks. Results from the HCP datasets demonstrate the possibility to reliably identify the language processing circuits, as well as the ten major resting-state circuits. Additionally, in the Fibercup phantom validation, we showed that the proposed model can successfully recover connections missed by tractography algorithms, and performed better than a recent state-of-the-art joint modeling algorithm^45^.

We note that the objective function of the network model can have a significant impact on the topology of the estimated joint flow network. In this paper, the *min-max* objective function was chosen to balance the flow in accordance with the strength of structural connectivity. Therefore, the final flow network represents the structural circuits under the constraints imposed by the functional connectivity data. Other objective functions may lead to complementary information and will be investigated in future work.

The proposed approach provides a novel and effective way to estimate function-specific structural connectivity in individual subjects (vs. group analysis). This may be useful in a wide range of neuroscience and clinical applications, such as the investigation of patterns of dysfunction in communication disorders (e.g., dyslexia), or neurodegeneration (e.g., Alzheimer’s disease, amyotrophic lateral sclerosis (ALS), Parkinson’s disease), where certain functions (e.g., language, memory, motor) are known to be affected. More specifically, applications of our method to clinical studies could lead to improved

- *Identification of connectivity alterations in brain diseases:* Beyond the numerous applications in which either functional connectivity patterns, or diffusion properties are compared between healthy controls and patients with neurological or psychiatric disorders^89–99^, the proposed approach uniquely enables the use of a new joint structural-functional “feature space”. By detecting differences in information flow (i.e., the combination of functional and structural connectivity, as demonstrated in Fig. 4 for instance), one may better understand the functional disruptions, and their associated neurobiological substrate (white matter pathways), which could ultimately lead to novel biomarkers. Moreover, if a specific function is known to be affected in a brain disorder (e.g., motor network in ALS), our method enables improved extraction of the corresponding white matter pathways, thereby facilitating the discovery of alterations which may not be identified by diffusion MRI tractography only. Of course, the drawback of this approach is that anatomical connections which do not support that specific brain function will not be tested and potential alterations could therefore be missed. In this situation, using resting-state fMRI in our model may be a better option, allowing the investigation of structural networks associated with all major brain functions.
- *Clinical outcome prediction:* Several prior works^100–102^ have demonstrated the ability to improve the prediction of clinical assessment scales or behavioral outcomes such as memory, using fMRI or dMRI. The additional information provided by our information flow network, or properties of a function-specific structural network, may lead to increased accuracy of such predictions.
- *Patients stratification:* The ability to separate structural networks/pathways based on the specific function they support offer new and rich information that could help identify patients subgroups in heterogeneous disorders such as schizophrenia^103–105^. We envision that new machine learning or classification algorithms may be able to take advantage of such information to improve their classification performance.
- *Longitudinal studies and assessment of treatment effects:* fMRI and dMRI have been widely used to monitor disease progression, or the effects of treatments in neurodegenerative disorders such as Alzheimer’s disease or ALS^101,106–108^. We believe that our approach might provide new information (joint flow network) or more specific data (enhanced structural connectivity mapping by incorporating fMRI data) which could help detect longitudinal changes, especially in disorders where the effect size of imaging biomarkers can be small, particularly in the early stages of the disease.
- *Pre-operative mapping and targeting intervention:* MRI has been utilized to provide pre-operative functional brain mapping, and help guide neurosurgical planning, especially to identify and avoid areas during surgical resection, that provide essential functions such as motor and language^109–113^. With the proposed scheme, both cortical/sub-cortical areas and associated white matter pathways which must be avoided could be identified simultaneously. Similarly, our approach could help other clinical interventions where localization of a functional area or white matter fiber bundle is critical, such as placement deep brain stimulation surgery, or transcranial magnetic stimulation (TMS).
  By identifying targeted structural sub-networks supporting distinct functions, our model may also help focus and improve advanced analyses in the following ways:
- Increase statistical power in graph-theoretic network analysis: Whole brain network analysis is useful to capture the interactions between several circuits and brain areas for cognitive, behavioral or motor functions114–116. Using prior knowledge about those functions, targeting only specific brain circuits identified with our proposed method could enable the detection of smaller effects.
- Improve the efficiency of time consuming investigations such as effective connectivity mapping: Computing effective connectivity is computationally expensive117,118. This could be partly overcome by performing effective connectivity estimation only for the connections of interest (i.e., for a specific brain function and associated structural network).

Our joint flow network model constitutes a useful tool to explore the relationship between functional and structural connectivity^9–21^. It could also be adapted to directly leverage effective connectivity information, from dynamic causal modeling or Granger causality^119–126^, and estimate the directionality of information flow. Finally, recent work^127^ has demonstrated that many tractography algorithms systematically identify a significant number of invalid white matter pathways, while (although to a far lesser extent) missing existing tracts. Such findings have serious implications for clinical applications such as neurosurgical planning^111^, and our proposed method may help mitigate the number of false-negative tracts. Future work will also focus on the generalization of the correction term *P*_*l*_, so it can take negative values and therefore minimize the number of false-positive tracts.

## Methods

### Datasets

Two different datasets were utilized in this study: Twenty-five subjects from the Human Connectome Project (HCP) Young Adult study^56,57^, and the realistic Fibercup diffusion phantom^58–60^.

Diffusion MRI, task-fMRI and resting-state fMRI HCP Open Access data were used to estimate the language processing and resting-state circuits (Section 2.2). It is publicly available from the ConnectomeDB database [https://db.humanconnectome.org]. All data were acquired on a customized Siemens 3T Connectome Skyra scanner with the following parameters: dMRI was obtained with 1.25 mm isotropic voxels, TR/TE = 5520/89.5 ms, three b-values of 1000, 2000 and 3000 s/mm^2^ and 90 directions per b-value with 18 additional b = 0 volumes^128^. Task and resting-state fMRI was obtained with 2 mm isotropic voxels, TR/TE = 720/33.1 ms, with respectively two runs of 3:57 min each, and four runs of 14:33 min each^66,75^.

The Fibercup data provides diffusion MRI data and the ground truth fibers layout, which can be used for validation. It is publicly available from the Tractometer website (http://www.tractometer.org/original_fibercup/data/) from the Sherbrooke Connectivity Imaging Lab. Ground truth for seven fiber bundles (Fig. 8(a)) and centers of the sixteen regions of interest (Fig. 8(c)) are available. The phantom simulates a coronal section from a human brain, and is suitable for evaluating connectivity estimation algorithms. The dMRI data of the phantom was obtained with 3 mm isotropic voxels, TR/TE = 5000/102 ms, and 64 directions uniformly distributed over the sphere. The data with b-value of 2000s/mm^2^ was used in our experiments.

**Figure 12:**
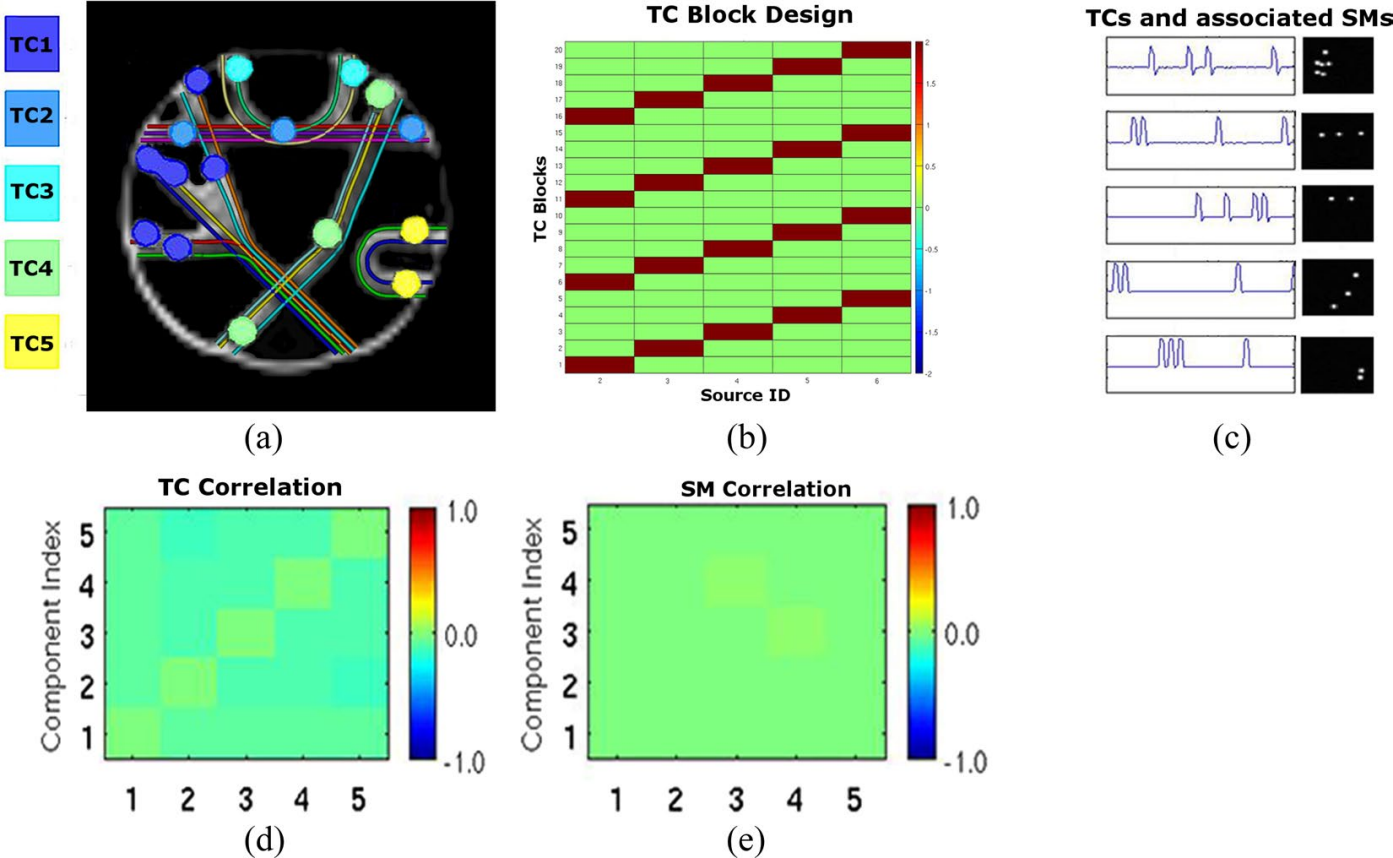
fMRI simulation for the Fibercup phantom: Five functional networks, corresponding to the structural connectivity pattern of the Fibercup phantom, were created to share similar fMRI time courses (e.g., TC1), as shown in panel (a). Nodes from the same network have the same color. A simple block design (Panel b) was used to generate BOLD signal time courses (TC) for each functional network (Panel c) by convolution with the hemodynamic function. Panels (d) and (e) show the correlations between base time courses and activation spatial maps (SM), and illustrate their independence. Panel (a) is adapted from Fig. 4 in Fillard *et al*.^58^.

#### Simulation of fMRI data

The fMRI BOLD signal for the Fibercup phantom is synthesized 100 times using the SimTB Toolbox^88^, and using the spatial patterns shown in Fig. 12(a). The images are generated with size 64 × 64 × 3, with 2 mm isotropic voxels, TR = 72 ms, and 1200 time points (similar to the HCP fMRI acquisition parameters). For each of the 100 realizations, the spatial coverage of each region is randomly and independently scaled by a factor (between 0.8 and 1.2), drawn from a uniform distribution to simulate structural variability. For regions belonging to the same circuit (e.g., P8 and P10), the BOLD signal shares the same activation pattern (block design) as illustrated in Fig. 12. Each time course consists of 20 ON time-blocks of 30 TRs and 20 OFF time-blocks of the same length. Unique random events for each network were added for each TR with a probability of 0.2 to introduce variability in the signal. Figure 12(c) shows the five base BOLD time courses allocated to the five networks. In Fig. 12(d) and (e), low correlations between base time courses and activation spatial maps illustrate their independence. Additionally, Rician noise was added to the BOLD signals.

### Data processing

#### Diffusion MRI data

The HCP dMRI data was first preprocessed following the default HCP preprocessing pipeline (v3.4)^129^, which includes correction for distortions and motion. Preprocessing is not needed for the Fibercup phantom data. After preprocessing, the Diffusion Toolkit/TrackVis [http://trackvis.org] was employed for diffusion tensor estimation and tractography^130^. The normalized streamline count between brain regions defined from the Desikan-Killiany brain parcellation [https://surfer.nmr.mgh.harvard.edu/fswiki/CorticalParcellation] was used to define structural connectivity, i.e., link capacity in the network, denoted as *D*_*l*_ in the network model described in Section 2.

#### Task and resting-state fMRI data

The HCP task-fMRI data was first processed following the HCP “fMRIVolume” pipeline (v3.4)^129^, which includes gradient unwrapping, motion/distortion correction, registration to structural scan, nonlinear registration into MNI152 space, and intensity normalization. Subsequently, spatial smoothing and activation maps generation using the general linear model (GLM) implemented in FSL’s FILM (FMRIB’s Improved Linear Model with autocorrelation)^55^ were performed. Additional details about the HCP “fMRIVolume” pipeline can be found in Barch *et al*.^66^. When computing the “average” Pearson correlation between pairs of brain areas (each with multiple fMRI time courses), the Fisher z-transformation was used before and after averaging correlation coefficients. For task-fMRI, ${R}_{i}^{m}$ is defined as the average value from the maps of regression parameter estimates (story and math tasks) within each cortical/sub-cortical area.

The HCP resting-state fMRI was also processed following the HCP “fMRIVolume” pipeline (v3.4)^129^. Spatial independent component analysis (spatial ICA) was conducted utilizing FSL melodic and FIX^53,54,131^. Subsequently, we used a recently proposed statistical method^132^ to identify meaningful (i.e., not noise) ICA components. The average magnitude of a spatial map *m* in a region *i* is interpreted as the functional activation of that region for mode (resting-state network) *m*, and is denoted as ${R}_{i}^{m}$, in the network model. Before calculating the average functional activation, the spatial maps were refined by masking with the Desikan-Killiany brain parcellation. After the average functional activation is computed, regions were further classified into either active or inactive for each individual resting-state network (mode) using K-means^132,133^. ${R}_{i}^{m}$ is set to zero for inactive regions.

## Availability of material and data

The datasets analyzed for this study are available from the Human Connectome Project (Open Access Data) ConnectomeDB database and from the Tractometer website from the Sherbrooke Connectivity Imaging Lab (for the phantom data). All connectivity matrices and numerical tables presented in this manuscript will be provided upon request to the authors. The source code will also be made freely available at http://www.cmrr.umn.edu/ downloads/.

## Electronic supplementary material

Supplementary Material
