# Supplementary material for: Function-specific and Enhanced Brain Structural Connectivity Mapping via Joint Modeling of Diffusion and Functional MRI
Source: Sci Rep. 2018 Mar 16;8:4741. doi: 10.1038/s41598-018-23051-9 (PMC5856752; doi:10.1038/s41598-018-23051-9)
Supplement: Supplementary file 1 — Supplementary Material [file 41598_2018_23051_MOESM1_ESM.pdf]

Function-specific and Enhanced Brain Structural Connectivity Mapping via Joint Modeling of Diffusion and Functional MRI

Shu-Hsien Chu[1], Keshab K. Parhi[1], and Christophe Lenglet[2,*]

[1]Electrical and Computer Engineering Department, University of Minnesota, Minneapolis, 55455, USA
[2]Center for Magnetic Resonance Research, University of Minnesota, Minneapolis, 55455, USA
[*]clenglet@umn.edu

Supplementary Material

A Supplementary Figures

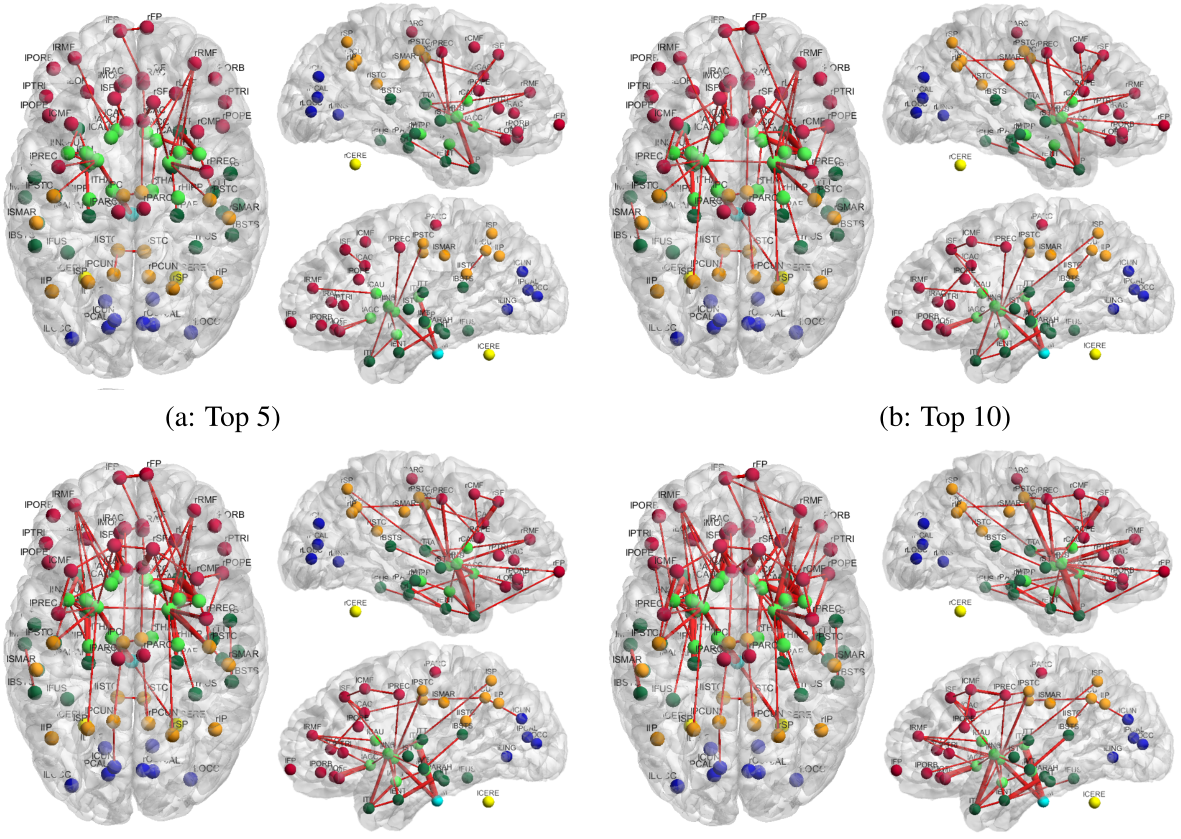

(a: Top 5) (b: Top 10)

(c: Top 15) (d: Top 20)

Figure S1. Consistency of the top (strongest) **structural connections**, estimated using tractography streamlines count, is shown in panels a (top 5), b (top 10), c (top 15) and d (top 20). The graphs are generated by selecting the top 5, 10, 15 and 20 structural connections from each individual subject. The edges' width, for each connection, represents the consistency across subjects, i.e., the frequency of identification across all subjects for a given threshold (5,10,15,20): The thicker the edge is, the more consistently the link is identified as a top-ranked connection across individuals. The nodes color represents the anatomical location: red for frontal, orange for parietal, blue for occipital, green for temporal, bright green for sub-cortical, yellow for cerebellum and bright blue for brainstem. Abbreviations for region labels are provided in Table S1.

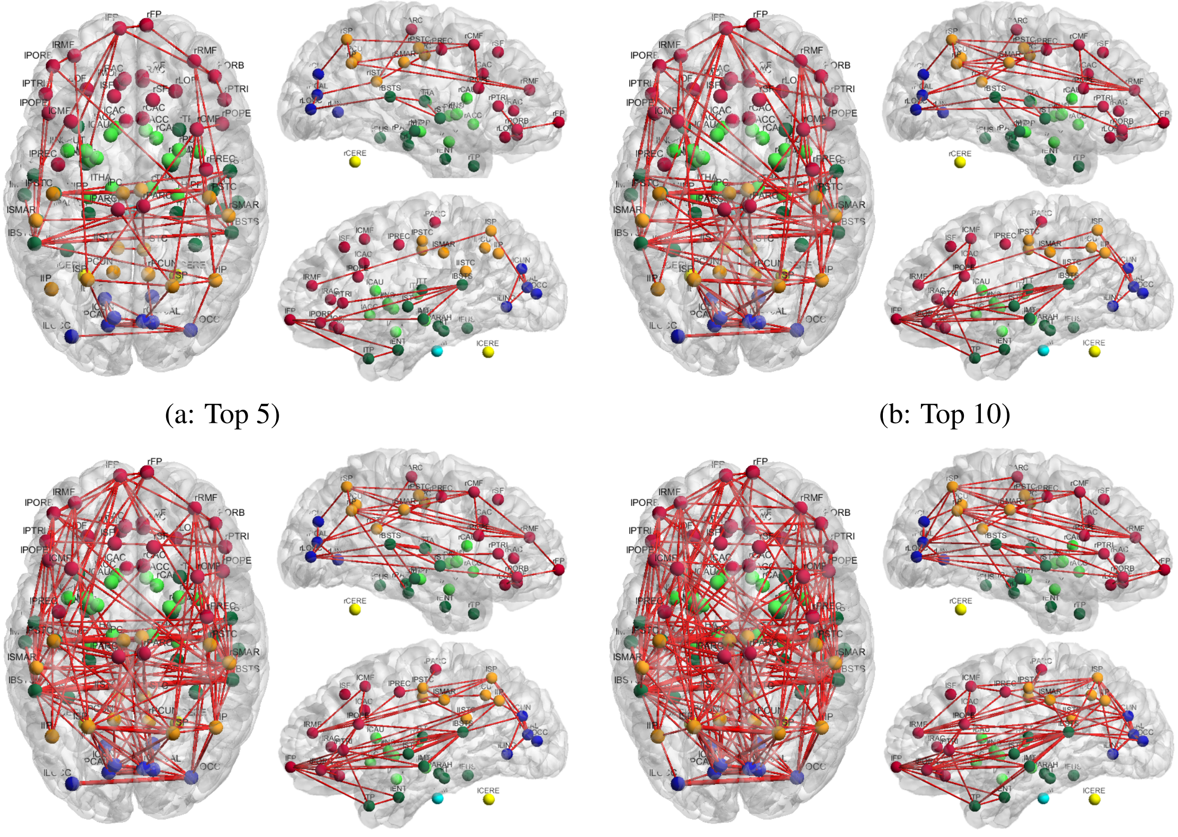

(a: Top 5) (b: Top 10)

(c: Top 15) (d: Top 20)

Figure S2. Consistency of the top (strongest) **functional connections** of the language processing areas, estimated using task-fMRI, is shown in panels a (top 5), b (top 10), c (top 15) and d (top 20). The graphs are generated by selecting the top 5, 10, 15 and 20 structural connections from each individual subject. The color code and labels are identical to Fig. S1.

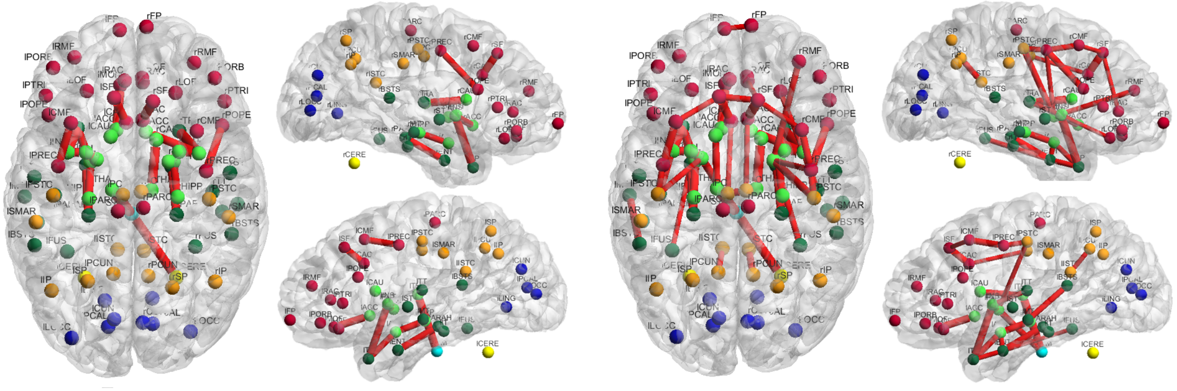

(a: Top 20) (b: Top 50)

Figure S3. Top 20 (a) and 50 (b) **structural connections**, with the strongest mean normalized connectivity (across subjects). Edges' width is proportional to mean connectivity value across subjects. The color code and labels are identical to Fig. S1.

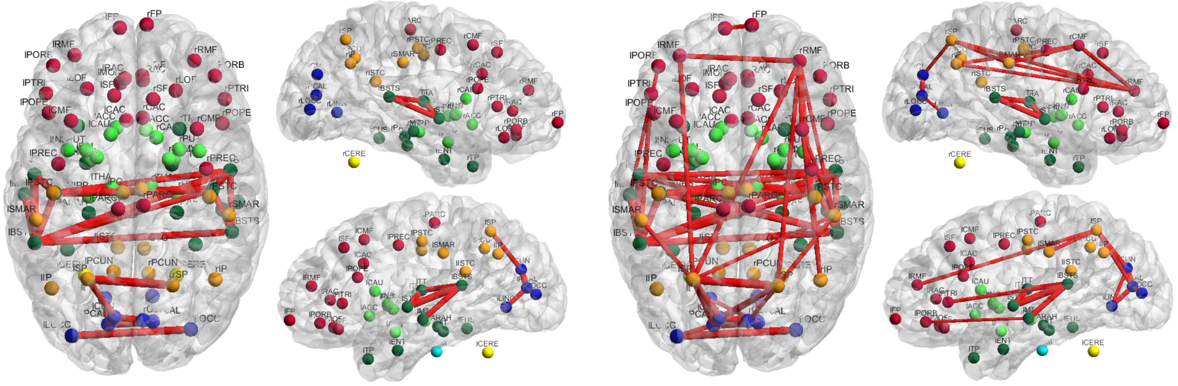

(a: Top 20) (b: Top 50)

Figure S4. Top 20 (a) and 50 (b) **functional connections** of the language processing areas, with the strongest mean normalized connectivity (across subjects). Edges' width is proportional to mean connectivity value across subjects. The color code and labels are identical to Fig. S1.

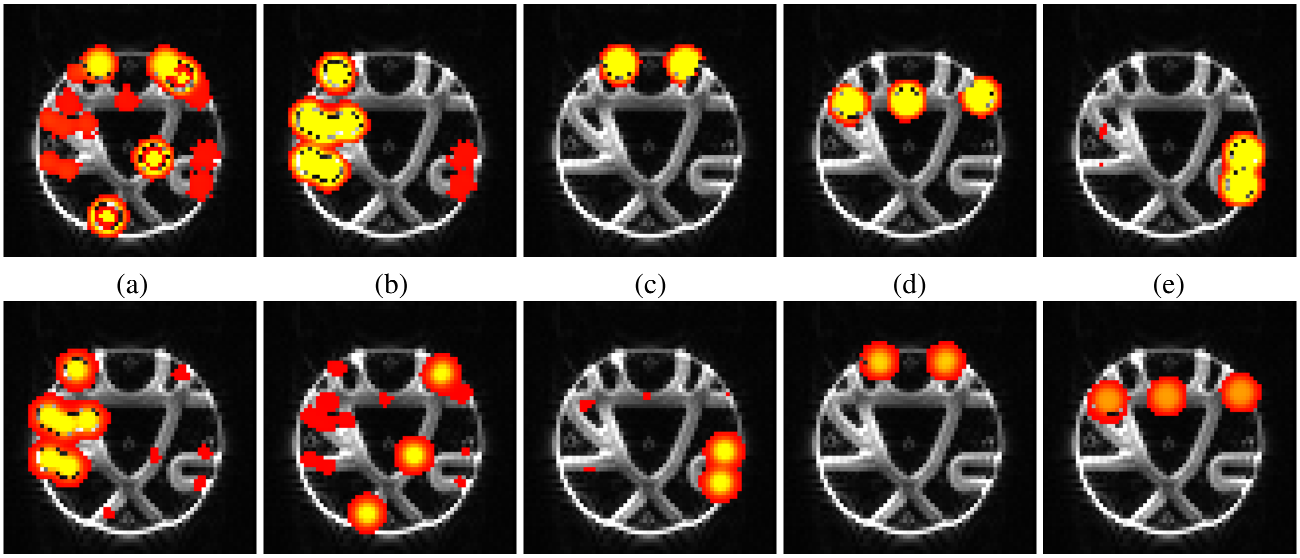

(a) (b) (c) (d) (e)

(f) (g) (h) (i) (j)

Figure S5. ICA spatial maps of two (top and bottom rows) representative realizations of the simulated fMRI data: After simulation of the fMRI signal within each of the sixteen end points (P1,...,P16), as described in Section 4 (Main Article), an ICA analysis enables the identification of the 5 sub-networks.

B Supplementary Section: Distributions of Connectivity Values

This section provides complementary information about the range of connectivity values, and their variability across subjects.

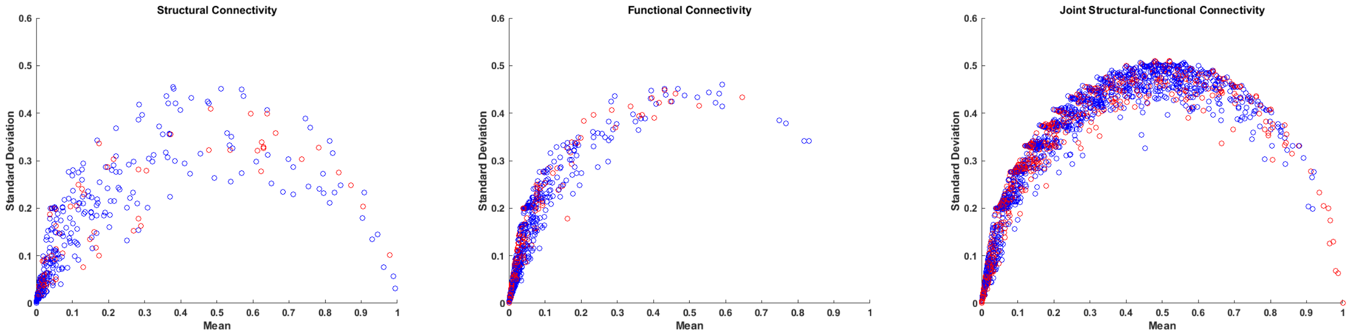

Figure S6. Scatter plots of the mean connectivity strength (x axis) and corresponding standard variation (y axis) across the twenty five subjects. The data is normalized for each subject, by the maximum connectivity value over all edges, in order to mitigate possible bias from a few subjects, and to ensure that connectivity values follow comparable distributions. Left panel represents the structural connectivity only (not function-specific). Center panel represents the functional connectivity only for the language processing task. Right panel represents the joint structural-functional connectivity for the language processing task (proposed model). Each point represents a specific connection between a pair of brain areas. The red points represent connections with at least one node in the temporal cortex, where major language processing areas are located

.

We note that the distribution of anatomical connectivity values shows few strong (high mean values) and consistent (low standard deviation values) connections that are known to exist from neuroanatomy, such as pathways between the putamen and pallidum (bilateral), brainstem and thalamus (bilateral), insula and temporal pole[1]. Connections with intermediate strength, which varies moderately from subject to subject were also identified, e.g., pathways between the amygdala and entorhinal cortex (bilateral), or between the pallidum and pre-/post-central gyri. Finally, many consistently weak connections were also found. Although some of these connections may represent real pathways with less dense white matter, it is also possible that they represent false-positive pathways, thereby reinforcing the need for joint structural-functional brain networks models to minimize such effects.

Functional connectivity values do not reach large values, as they are defined by Pearson correlation coefficient between fMRI time series, with a maximum of about 0.8. A few strong connections with moderate variability are identified. They include several connections with the left banks of the superior temporal sulcus, as well as connections between the left and right superior parietal cortices, which have been shown to play an important role[2] in language processing.

Joint structural-functional (flow) connectivity values are overall higher (more points, especially towards the right side of the plot, than for structural or functional connectivity), with more connections involving the temporal areas (red points). This is explained by the ability of the model to recover connections with under-estimated structural connectivity, via the incorporation of functional connectivity information from the language processing task fMRI data. A few connections exhibit very high connectivity (flow) value and low variability: they include pathways between the banks of the superior temporal sulcus and inferior parietal cortex (bilateral), between the superior and transverse temporal cortices (bilateral), and between the insula and transverse temporal cortex (bilateral). Variability also increases for connections with moderate strength. Finally, more connections also exhibit very low (close to zero) connectivity and variability: these likely correspond to false-positive pathways which are better detected by combining structural and functional connectivity information.

C Supplementary Tables

Table S1. Brain parcellation (Desikan-Killiany) of cortical/sub-cortical areas, and corresponding abbreviations. For each area, the first letter (lower case) is used through the manuscript to denote location in the (l)eft and (r)ight hemisphere, e.g., lCERE and rCERE

(Sub-)Cortical areas	Abbr.	(Sub-)Cortical areas	Abbr.
Brainstem	STEM	Paracentral	PARC
Cerebellum	CERE	Pars Opercularis	POPE
Thalamus	THA	Pars Orbitalis	PORB
Caudate	CAU	Pars Triangularis	PTRI
Putamen	PUT	PeriCalcarine	PCAL
Pallidum	PAL	Postcentral	PSTC
Hippocampus	HIPP	Posterior Cingulate	PC
Amygdala	AMY	Precentral	PREC
Accumbens	ACC	Precuneus	PCUN
Banks Sup. Temp. Sulc.	BSTS	Rostral Anterior Cingulate	RAC
Cuneus	CUN	Rostral Middle Frontal	RMF
Entorhinal	ENT	Superior Frontal	SF
Fusiform	FUS	Superior Parietal	SP
Inferior Parietal	IP	Superior Temporal	ST
Inferior Temporal	IT	Supramarginal	SMAR
Isthmus Cingulate	ISTC	Frontal Pole	FP
Lateral Occipital	LOCC	Temporal Pole	TP
Lateral Orbitofrontal	LOF	Transverse Temporal	TT
Lingual	LING	Insula	INS
Medial Orbitofrontal	MOF	Caudal Anterior Cingulate	CAC
Middle Temporal	MT	Caudal Middle Frontal	CMF
ParaHippocampal	PARAH		

Table S2. Top 50 connections with strongest average **structural connectivity**

Connection	Mean	Std. Dev.	Connection	Mean	Std. Dev.
(R)Putamen - (R)Pallidum	0.994	0.032	(L)Thalamus Proper - (L)Caudate	0.691	0.24
(L)Thalamus Proper - Brainstem	0.988	0.06	(R)Caudal Middle Frontal - (R)Precentral	0.679	0.313
(R)Temporal Pole - (R)Insula	0.979	0.105	(R)Postcentral - (R)Precentral	0.658	0.245
(L)Putamen - (L)Pallidum	0.958	0.091	(R)Entorhinal - (R)Temporal Pole	0.646	0.364
(R)Caudal Anterior Cingulate - (R)Superior Frontal	0.938	0.164	(R)Pallidum - (R)Rostral Middle Frontal	0.632	0.409
(R)Thalamus Proper - (R)Caudate	0.931	0.150	(R)Amygdala - (R)Temporal Pole	0.627	0.402
(L)Accumbens - (L)Medial Orbitofrontal	0.909	0.239	(L)Superior Frontal - (R)Superior Frontal	0.625	0.323
(L)Temporal Pole - (L)Insula	0.897	0.219	(L)Postcentral - (L)Precentral	0.622	0.262
Brainstem - (R)Cerebellum	0.893	0.195	(R)Amygdala - (R)Entorhinal	0.611	0.337
(L)Entorhinal - (L)ParaHippocampal	0.865	0.262	(L)Transverse Temporal - (L)Insula	0.608	0.344
(R)Entorhinal - (R)ParaHippocampal	0.836	0.286	(R)Entorhinal - (R)Fusiform	0.606	0.334
(L)Caudal Anterior Cingulate - (L)Superior Frontal	0.829	0.269	(R)Transverse Temporal - (R)Insula	0.604	0.29
(R)Putamen - (R)Insula	0.815	0.325	(L)Amygdala - (L)Entorhinal	0.597	0.326
(L)Putamen - (L)Insula	0.814	0.247	(L)Isthmus Cingulate - (L)Precuneus	0.590	0.309
(R)Accumbens - (R)Insula	0.808	0.346	(L)Entorhinal - (L)Temporal Pole	0.582	0.406
(L)Hippocampus - (L)Amygdala	0.801	0.23	(R)Pallidum - (R)Precentral	0.567	0.437
(R)Pars Opercularis - (L)Precentral	0.782	0.294	(L)Pallidum - (L)Postcentral	0.565	0.452
(L)Caudal Middle Frontal - (L)Precentral	0.774	0.253	(L)Caudal Middle Frontal - (L)Superior Frontal	0.543	0.276
(R)Hippocampus - (R)Amygdala	0.766	0.259	(L)Caudal Anterior Cingulate - (L)Posterior Cingulate	0.523	0.355
(L)Amygdala - (L)Temporal Pole	0.766	0.338	(R)Lateral Orbitofrontal - (R)Insula	0.518	0.267
(L)Pallidum - Brainstem	0.752	0.374	(R)Rostral Anterior Cingulate - (R)Superior Frontal	0.517	0.329
(R)Pallidum - (R)Postcentral	0.741	0.393	(R)Caudal Anterior Cingulate - (R)Posterior Cingulate	0.516	0.337
(L)Posterior Cingulate - (R)Posterior Cingulate	0.733	0.261	(R)Caudal Middle Frontal - (R)Superior Frontal	0.513	0.358
(L)Banks Sup. Temp. Sulc. - (L)Middle Temporal	0.727	0.316	(L)Entorhinal - (L)Fusiform	0.512	0.33
(R)Isthmus Cingulate - (R)Precuneus	0.697	0.299	(L)Frontal Pole - (R)Frontal Pole	0.51	0.455

Table S3. Top 50 connections with strongest average **functional connectivity** (language processing task)

Connection	Mean	Std. Dev.	Connection	Mean	Std. Dev.
(L)Banks Sup. Temp. Sulc. - (L)Superior Temporal	0.836	0.281	(L)Rostral Middle Frontal - (R)Rostral Middle Frontal	0.507	0.332
(L)Superior Parietal - (R)Superior Parietal	0.822	0.276	(L)Superior Temporal - (R)Transverse Temporal	0.493	0.443
(L)Transverse Temporal - (R)Transverse Temporal	0.8	0.36	(R)Caudal Middle Frontal - (R)Rostral Middle Frontal	0.484	0.427
(L)PeriCalcarine - (R)PeriCalcarine	0.761	0.381	(L)PeriCalcarine - (L)Superior Parietal	0.483	0.433
(L)Cuneus - (L)PeriCalcarine	0.750	0.399	(L)Superior Parietal - (R)Rostral Middle Frontal	0.476	0.341
(L)Lateral Occipital - (R)Lateral Occipital	0.742	0.358	(L)Rostral Middle Frontal - (L)Superior Parietal	0.469	0.427
(L)Banks Sup. Temp. Sulc. - (R)Superior Temporal	0.721	0.377	(L)Lateral Occipital - (L)PeriCalcarine	0.462	0.421
(L)Superior Temporal - (L)Transverse Temporal	0.71	0.382	(L)Banks Sup. Temp. Sulc. - (L)Pars Triangularis	0.459	0.436
(L)Banks Sup. Temp. Sulc. - (L)Transverse Temporal	0.688	0.424	(R)Rostral Middle Frontal - (R)Superior Parietal	0.454	0.432
(L)Cuneus - (R)Cuneus	0.685	0.393	(L)Cuneus - (L)Lingual	0.448	0.438
(L)Banks Sup. Temp. Sulc. - (L)Middle Temporal	0.682	0.372	(R)Superior Parietal - (R)Supra Marginal	0.444	0.428
(L)Banks Sup. Temp. Sulc. - (R)Banks Sup. Temp. Sulc.	0.681	0.443	(L)Superior Temporal - (R)Banks Sup. Temp. Sulc.	0.441	0.434
(R)Superior Temporal - (R)Transverse Temporal	0.662	0.42	(L)Superior Parietal - (L)Supra Marginal	0.439	0.4
(R)Banks Sup. Temp. Sulc. - (R)Superior Temporal	0.657	0.433	(R)Caudal Middle Frontal - (R)Inferior Parietal	0.437	0.419
(L)Cuneus - (R)PeriCalcarine	0.645	0.39	(L)Lingual - (R)PeriCalcarine	0.418	0.436
(L)Transverse Temporal - (R)Superior Temporal	0.619	0.433	(R)Inferior Parietal - (R)Rostral Middle Frontal	0.416	0.432
(R)Banks Sup. Temp. Sulc. - (R)Transverse Temporal	0.615	0.422	(R)Cuneus - (R)Superior Parietal	0.415	0.434
(L)Cuneus - (L)Superior Parietal	0.591	0.43	(L)Frontal Pole - (R)Frontal Pole	0.405	0.425
(L)Lingual - (L)PeriCalcarine	0.567	0.434	(L)PeriCalcarine - (R)Superior Parietal	0.398	0.414
(L)Banks Sup. Temp. Sulc. - (R)Transverse Temporal	0.562	0.461	(R)Rostral Middle Frontal - (R)Supra Marginal	0.386	0.43
(L)Cuneus - (R)Superior Parietal	0.549	0.425	(R)Caudal Middle Frontal - (R)Supra Marginal	0.378	0.407
(R)Cuneus - (R)PeriCalcarine	0.541	0.466	(L)PeriCalcarine - (R)Lingual	0.376	0.429
(L)Superior Temporal - (R)Superior Temporal	0.538	0.446	(L)Superior Parietal - (R)Supra Marginal	0.373	0.393
(L)Transverse Temporal - (R)Banks Sup. Temp. Sulc.	0.521	0.444	(L)Middle Temporal - (L)Pars Orbitalis	0.367	0.411
(L)PeriCalcarine - (R)Cuneus	0.516	0.447	(R)Lingual - (R)PeriCalcarine	0.359	0.418

Table S4. Top 50 connections with strongest average **joint structural-functional (flow) connectivity** (language processing task)

Connection	Mean	Std. Dev.	Connection	Mean	Std. Dev.
(L)Banks Sup. Temp. Sulc. - (L)Inferior Parietal	1	0	(L)Caudate - (R)Caudate	0.911	0.268
(L)Superior Temporal - (L)Transverse Temporal	1	0	(R)Cerebellum - (R)Precentral	0.904	0.213
(L)Transverse Temporal - (L)Insula	1	0	Brainstem - (L)Superior Frontal	0.89	0.308
(R)Superior Temporal - (R)Transverse Temporal	1	0	(R)Cerebellum - (R)Thalamus Proper	0.88	0.332
(R)Transverse Temporal - (R)Insula	1	0	(L)Cerebellum - (L)Lingual	0.88	0.332
(R)Banks Sup. Temp. Sulc. - (R)Inferior Parietal	0.991	0.044	(R)Middle Temporal - (R)Temporal Pole	0.88	0.332
(L)Postcentral - (L)Transverse Temporal	0.985	0.054	(R)Accumbens - (R)Insula	0.88	0.332
(L)Supra Marginal - (L)Transverse Temporal	0.974	0.130	(L)Superior Frontal - (R)Caudal Anterior Cingulate	0.865	0.330
(R)Postcentral - (L)Transverse Temporal	0.966	0.119	(L)Pars Opercularis - (L)Superior Temporal	0.864	0.317
(R)Supra Marginal - (R)Transverse Temporal	0.964	0.178	(R)Caudate - (L)Medial Orbitofrontal	0.850	0.322
(R)Thalamus Proper - (R)Pallidum	0.96	0.2	(L)Putamen - (L)Entorhinal	0.848	0.336
(L)Banks Sup. Temp. Sulc. - (L)Middle Temporal	0.96	0.2	(R)Thalamus Proper - (R)Entorhinal	0.848	0.358
(R)Banks Sup. Temp. Sulc. - (R)Superior Temporal	0.96	0.2	(R)Pars Opercularis - (R)Superior Temporal	0.844	0.333
(R)Precentral - (R)Superior Temporal	0.950	0.204	(L)Cerebellum - (L)Thalamus Proper	0.84	0.374
(R)Banks Sup. Temp. Sulc. - (R)Middle Temporal	0.935	0.231	(L)Thalamus Proper - (L)Pallidum	0.84	0.374
(R)Cerebellum - (R)Superior Parietal	0.922	0.189	(L)Thalamus Proper - (R)Thalamus Proper	0.84	0.374
(L)Cerebellum - (R)Cerebellum	0.92	0.277	(L)Banks Sup. Temp. Sulc. - (L)Superior Temporal	0.84	0.374
(L)Caudal Middle Frontal - (L)Pars Opercularis	0.92	0.277	(L)Middle Temporal - (L)Temporal Pole	0.84	0.374
(L)Precentral - (L)Superior Temporal	0.92	0.277	(R)Postcentral - (R)Superior Temporal	0.84	0.374
(L)Banks Sup. Temp. Sulc. - (L)Supra Marginal	0.92	0.277	Brainstem - (L)Hippocampus	0.837	0.373
(R)Cerebellum - (R)Lingual	0.92	0.277	(R)Pallidum - (R)Amygdala	0.837	0.35
(R)Caudal Middle Frontal - (R)Pars Opercularis	0.92	0.277	(L)Cerebellum - (L)Precentral	0.837	0.304
(R)Banks Sup. Temp. Sulc. - (R)Supramarginal	0.92	0.277	Brainstem - (R)Rostral Middle Frontal	0.833	0.347
(R)Amygdala - (R)Insula	0.92	0.277	Brainstem - (L)Rostral Middle Frontal	0.827	0.363
(L)Postcentral - (L)Superior Temporal	0.915	0.277	(L)Putamen - (R)Putamen	0.825	0.366

References

1. Jakab, A., Molnár, P. P., Bogner, P., Béres, M. & Berényi, E. L. Connectivity-based parcellation reveals interhemispheric differences in the insula. *Brain topography* **25**, 264–271 (2012).
2. Segal, E. & Petrides, M. The anterior superior parietal lobule and its interactions with language and motor areas during writing. *Eur. J. Neurosci.* **35**, 309–322 (2012).
